# Synthetic microbial community SMC-L1 optimizes flavor chemistry in reduced salt soy sauce via targeted metabolic reprogramming

**DOI:** 10.3389/fmicb.2025.1701479

**Published:** 2025-11-12

**Authors:** Yuqi Gao, Lin Zhang, Yi Zhang, Jun Huang, Chongde Wu, Rongqing Zhou

**Affiliations:** Country College of Biomass Science and Engineering, Sichuan University, Chengdu, China

**Keywords:** soy sauce, *Tetragenococcus halophilus*, salt-reduction fermentation, microbial community, metabolic profile

## Abstract

The high sodium content in traditional soy sauce presents significant public health concerns, particularly related to hypertension and cardiovascular diseases. However, reducing salt content often disrupts microbial ecology and impairs flavor formation during fermentation. To overcome this challenge, we developed synthetic microbial communities (SynMCs) for reduced-salt (13% NaCl) moromi fermentation under traditional sun-brewing conditions. Using integrated multi-omics analyses, we identified an optimal consortium (SMC-L1) incorporating *Tetragenococcus halophilus* T10 as a key lactic acid bacterium alongside functional yeast strains. This defined community maintained fermentation stability while significantly enhancing flavor-relevant biochemical profiles. SMC-L1 inoculation markedly improved key quality parameters, increasing total nitrogen by 40.8% and amino acid nitrogen by 56.7%. Furthermore, it elevated critical metabolites including organic acids, particularly succinate, free amino acids, and short-chain esters. Network analysis revealed robust ecology-metabolite relationships: *Tetragenococcus* abundance correlated with succinate production and ester synthesis, while *Aspergillus* dynamics corresponded with free amino acid accumulation. These findings highlight how targeted microbial consortia can reprogram metabolic networks under salt-reduced conditions. From a food microbiology perspective, this study demonstrates that rational design of microbial communities can effectively decouple salt reduction from flavor deterioration in fermented foods. The metabolic pathways observed, particularly the anaerobic TCA cycle activity connecting *Tetragenococcus* to succinate accumulation, provides mechanistic insights into microbial adaptation to reduced-salt environments. This approach offers a viable strategy for developing healthier fermented products without compromising their sensory characteristics, advancing both fundamental knowledge and practical applications in food biotechnology.

## 1 Introduction

Hypertension poses a severe and growing public health challenge worldwide, affecting approximately 31.1% of the global adult population—around 1.39 billion people—in 2010 (Mills et al., 2020). Chronic high salt intake is a well-established risk factor for hypertension, cardiovascular disease, and osteoporosis. This problem is particularly relevant in East Asia, where hypertension prevalence exceeds 27.5% among populations with high soy sauce consumption. As an essential condiment, soy sauce is highly valued for its unique flavor and color (An et al., 2023). However, its high sodium content is directly linked to health risks, making salt reduction an urgent industry priority (He and Tan, 2024; Okada et al., 2018). Despite these concerns, the global soy sauce market continues to expand steadily, reaching over 270 billion RMB in 2024 and accounting for 12.8% of the global condiment market, highlighting its significant economic and culinary role and driving the development of innovative reduced-salt fermentation techniques. Fermentation processes are classified by moromi salt concentration and moisture: high-salt liquid-state fermentation (HLF; >18% w/w NaCl, semi-solid) and low-salt solid-state fermentation (LSF; <12% w/w NaCl, solid), which differ significantly in duration, mechanisms, and metabolites (Zhang et al., 2016). LSF typically employs a koji-to-brine ratio of 1:1.1–1:1.2, whereas HLF uses 1:1.8–1:2.5. Temperature control methods include traditional sun-brewing (ambient), constant-temperature (30 ± 2 °C), and multi-stage regimes. Traditional Chinese production relies on sun-brewing, where indigenous microbiota degrades proteins and starches to generate flavor compounds (e.g., amino acids, sugars, esters) (Liu et al., 2020; Zhao et al., 2023). Regional and seasonal microbial variations impart artisanal flavors but compromise production consistency and process efficiency. Modern industrial methods enhance reproducibility through controlled fermentation yet fail to replicate natural microbial complexity, often yielding organoleptically inferior products (Feng et al., 2024). Consequently, sun-brewing remains essential for achieving distinctive sensory profiles.

During natural fermentation, *Tetragenococcus halophilus* and *Zygosaccharomyces rouxii*, exhibit coordinated metabolic interplay wherein bacterial organic acids (e.g., acetic and lactic acid) can modulate yeast activity by inhibiting growth and influencing metabolic pathways (Cao et al., 2009). As established in prior studies, their growth follows a defined succession wherein the bacterial organic acids regulate yeast activity (Tanaka et al., 2012; Liang et al., 2019; Yang et al., 2017; Kusumegi et al., 1998). Specially, acetic and lactic acid produced by *T. halophilus* inhibit the growth of *Z. rouxii* and *Candida versatilis*. The inhibition of *Z. rouxii* by acetic acid involves three key mechanisms: suppression of respiratory activity, inhibition of cytochrome formation, and impairment of proton expulsion. Notably, the impairment of proton expulsion is critically linked to osmo-tolerance (Cao et al., 2009). To address this microbial antagonism, Zhang et al. (2024a) developed a sequential inoculation strategy using functional strains that increased volatile organic compounds (VOCs) in *moromi* by 216.88%. This demonstrates that modulating functional strain interactions effectively enhances reduced-salt soy sauce flavor. Synthetic microbial communities containing *Tetragenococcus halophilus*, *Zygosaccharomyces rouxii*, and other species promote key flavor compound synthesis, including 2-furanmethanol (caramel aroma) and phenethyl acetate (floral-honey aroma) (Devanthi et al., 2018). Integration of *Meyerozyma guilliermondii* further elevates compounds such as 4-hydroxy-2,5-dimethyl-3(2H)-furanone (caramel) and maltol (malty), while degrading biogenic amines in reduced-salt moromi (Singracha et al., 2017). Collectively, these findings provide a foundation for synthetic consortium development.

Despite the industry trend toward reduced-sodium soy sauce, salt reduction disrupts microbial equilibrium, potentially enabling pathogen proliferation (Hu et al., 2023) while impairing formation of critical flavor components [e.g., benzyl alcohol, 4-hydroxy-2,5-dimethyl-3(2H)-furanone], causing sensory deficits (Liu et al., 2023). Current reduction strategies include potassium-based alternatives (posing renal risks) and electrodialysis, causing 30–40% amino acid loss (Zheng et al., 2024). These conventional approaches incur significant limitations including high costs and flavor deterioration. By contrast, strategies leveraging core brewing microbiota to enhance the flavor of reduced-salt soy sauce offer greater promise. Hu et al. (2024) achieved moromi fermentation at 12% NaCl using *Weissella paramesenteroides* and *Bacillus amyloliquefaciens*, while Song et al. (2015) maintained typical flavor at 8% salt with *Torulaspora delbrueckii* and *Zygosaccharomyces rouxii*. Nevertheless, salt reduction specifically in traditional Chinese sun-brewing remains unexplored, a critical gap given this method’s important role in flavor development. Into sun-brewed moromi.

In this study, we employed a synthetic microbial community (SynMC) approach, defined as a consortium of three functional strains: one lactic acid bacterium (LAB) and two yeasts (*Z. rouxii* QH-25 and *C. versatilis* CGMCC 3790). The yeast strains were held constant to form a stable functional backbone, while the LAB component was systematically varied to construct three distinct SynMCs (designated SMC-L1, SMC-L2, and SMC-L3). The objective was to identify which LAB module, within this fixed framework, best sustains quality-related chemistry in a reduced-salt soy sauce (13% NaCl) moromi fermentation under traditional sun-brewed conditions. This design evaluates the feasibility of producing reduced-salt soy sauce compatible with traditional practices while preserving key sensory attributes. The performance of the SynMCs was assessed through comparative physicochemical, microbiome, and metabolomic analyses against a conventional high-salt (≥16% NaCl) control fermentation (HLF).

## 2 Materials and methods

### 2.1 Materials and reagents

Fresh koji samples were obtained from a single production workshop at Qianhe Condiment Co., Ltd. (Meishan City, Sichuan Province, China). The strains *Tetragenococcus halophilus* CGMCC 3792, *Tetragenococcus halophilus* T10, *Zygosaccharomyces rouxii* QH-25, and *Candida versatilis* CGMCC 3790 were previously isolated from soybean paste and moromi mash. These strains were identified, preserved, and applied in prior studies (Zhang et al., 2024b; Zhang et al., 2021; Cui et al., 2014). *Lactobacillus plantarum* 1.08 was obtained from Shanghai Difa Brewing Bio-Products Co., Ltd. (China). All yeast and lactic acid bacteria strains were activated in soy sauce culture medium according to Qi et al. (2022). Organic acid standards were sourced from Sigma-Aldrich (St. Louis, MO, United States). Amino acid mixed standards were purchased from membraPure GmbH (Germany). 2-Octanol (chromatographic grade), employed as an internal standard for gas chromatography–mass spectrometry (GC–MS), was obtained from Aladdin Reagent (Shanghai, China). Qualitative Filter Paper (Model 101) was produced by the Fushun Civil Affairs Filter Paper Factory (Fushun city, Liaoning province, China). All other reagents, including peptone, yeast extract powder, glucose, potassium dihydrogen phosphate, starch, sodium chloride, anhydrous magnesium sulfate, sodium hydroxide, hydrochloric acid, formaldehyde, copper sulfate, potassium sulfate, methyl red, bromocresol green, and silver nitrate, were of analytical grade and procured from Shudu Laboratory Equipment Co. (Chengdu, China).

### 2.2 Fermentation setup and conditions

Fresh koji from a single production batch (Qianhe Condiment Co., Ltd.) was used to ensure consistency. Moromi was prepared by mixing koji with brine at a 1:1.8 (w/w) ratio. We established four experimental groups: a high-salt control (16% NaCl) without inoculation, and three reduced-salt (13% NaCl) groups inoculated with different synthetic microbial communities (SynMCs). The three SynMCs shared a fixed yeast backbone (*Zygosaccharomyces rouxii* and *Candida versatilis*) and differed only in the LAB module: SMC-L1 contained *Tetragenococcus halophilus* T10; SMC-L2 contained *Lactobacillus plantarum* 1.08; SMC-L3 contained *T. halophilus* CGMCC 3792.

Based on the established microbial succession in moromi fermentation where lactic acid bacteria (LAB) precede yeasts (Yong and Wood, 1976), a sequential inoculation strategy was employed to mitigate microbial antagonism and promote aroma development, following the approach of Zhang et al. (2024b). Specifically, *Tetragenococcus halophilus* was inoculated on day 1 to initiate acidification, followed by *Zygosaccharomyces rouxii* on day 4 after initial salt and pH equilibration, and finally *Candida versatilis* on day 10.

All fermentations were carried out in 50 L ceramic vessels, which were sterilized with ethanol, and maintained at ambient temperature. Each group was prepared in biological triplicate; detailed sample assignments are listed in Table 1. Initial inoculum levels were 6.80 × 10^6^ CFU/g (*T. halophilus* CGMCC 3792), 5.23 × 10^6^ CFU/g (*T. halophilus* T10), 5.50 × 10^6^ CFU/g (*L. plantarum* 1.08), 6.72 × 10^6^ CFU/g (*Z. rouxii* QH-25), and 8.22 × 10^6^ CFU/g (*C. versatilis* CGMCC 3790). The moromi was stirred every 48 h during the first 15 days and every 120 h thereafter. After 5 months of fermentation, samples were harvested and stored at −20 °C for metabolic analyses and at −80 °C for microbial community profiling.

**Table 1:** Sample information.

Sample type	Sample name	Strains and addition time
Reduced salt grouping	SMC-L1	The addition time of *T. halophilus* T10 on day 1, then *Z. rouxii* QH-25 on day 4, and finally *C. versatilis* CGMCC 3790 on day 10
	SMC-L2	The addition time of *T. halophilus* CGMCC 3792 on day 1, then *Z. rouxii* QH-25 on day 4, and finally *C. versatilis* CGMCC 3790 on day 10
	SMC-L3	The addition time of *Lactobacillus plantarum* 1.08 on day 1, then *Z. rouxii* QH-25 on day 4, and finally *C. versatilis* CGMCC 3790 on day 10
High salt grouping	HS	According to traditional factory techniques

### 2.3 Sample preparation and physicochemical properties analysis

Salt levels are reported as % (w/w) NaCl of the total moromi, determined by chloride titration at 20–25 °C. In this work, experimental groups were controlled at setpoints: High-salt (HS) control and the salt-reduced groups at 13% NaCl. All reported ‘salt’ values refer to the NaCl mass fraction in the total moromi.

Moromi was filtered through qualitative filter Paper, then the filtrate was immediately stored at −20 °C for subsequent analysis of physicochemical properties and metabolites. The following physicochemical parameters were measured according to the referenced Chinese national standards: soluble solids (GB 18186–2000), sodium chloride (GB 18186–2000), total nitrogen (TN, GB 18186–2000), amino acid nitrogen (AAN, GB 18186–2000), total acidity (TA, GB 18186–2000), ammonium salt (GB 5009.39), and reducing sugars (RS, GB 5009.7–2016). For each determination, 5 mL aliquots of filtrate were diluted to 100 mL with deionized water. Ethanol content was determined by potassium dichromate colorimetry. Briefly, 5 mL of diluted sample was distilled, and 95 mL of distillate was collected in a volumetric flask and brought to 100 mL with distilled water. Then, a 5 mL aliquot of this solution was mixed with 5 mL of concentrated H₂SO₄ and 1 mL of 4% (w/v) K₂Cr₂O₇ in a colorimetric tube. The mixture was heated in a boiling water bath for 10 min, cooled to room temperature, and the absorbance was measured at 600 nm.

### 2.4 Organic acid analyzing

High-performance liquid chromatography (HPLC) was performed following a modified method from Zhang et al. (2021). In brief, 5 mL samples were vortex-mixed with 10 mL of 9 mM H₂SO₄ and subjected to ultrasonic extraction. The sample was vortexed for 1 min at 15-min intervals, for a total of four cycles. Following extraction, the mixture was centrifuged at 7,500 × g for 5 min, the supernatant was centrifuged at 12,000 × g for 10 min. A 3 mL aliquot of the final supernatant was applied to a pre-conditioned silica SPE cartridge (Chroclean SILICA, activated with 3 mL methanol and 3 mL ultrapure water). The eluate was passed through a 0.22-μm aqueous membrane into an HPLC vial. Analytes were identified by matching retention time to those of authentic standards and quantified using external standard calibration.

The HPLC analysis was conducted using an Avantor Hichrom OA-1000 organic acid column (9 μm, 300 mm × 6.5 mm); UV detector (wavelength 215 nm); mobile phase: 100% 9 mM H₂SO₄ solution; flow rate: 0.6 mL/min; injection volume: 10 μL; column oven temperature: 75 °C.

### 2.5 Amino acid analysis

Free amino acids were quantified using an automatic amino acid analyzer (A300, membraPure GmbH, Germany) following a modified protocol from Qi et al. (2022).

For sample preparation, 1 mL of soy sauce filtrate was diluted to 100 mL with 0.01 M HCl in a volumetric flask. The diluted samples were sonicated in an ice bath for 30 min, vortexed briefly, and centrifuged at 12,000 × g for 10 min. Subsequently, a 5 mL aliquot of supernatant was mixed with 5 mL of 10% (w/v) sulfosalicylic acid to precipitate protein at 4 °C for at least 2 h, followed by recentrifugation at 12,000 × g for 10 min. The resulting supernatant was filtered through a 0.22-μm aqueous syringe filter, and the filtrate solution was directly injected into the amino acid analyzer for quantification.

### 2.6 Volatile compound analysis

Volatile compounds were analyzed by headspace solid-phase microextraction coupled with gas chromatography–mass spectrometry (HS-SPME-GC–MS). A 1.0 mL soy sauce sample was mixed with 0.2 g NaCl and 10 μL of 2-octanol (internal standard) in a 20 mL headspace vial. After equilibration at 60 °C for 15 min in a thermostatic water bath, volatile compounds were extracted by exposing a DVB/CAR/PDMS fiber (Supelco, Bellefonte, PA, United States) to the vial headspace for 45 min at 60 °C.

Adsorbed compounds were thermally desorbed at 250 °C for 5 min in the injection port of a GC–MS system (Thermo TRACE 1300 gas chromatograph coupled to a TSQ 9000 triple-quadrupole mass spectrometer; Waltham, MA, United States). Chromatographic separation was performed on a DB-WAX capillary column (60 m × 0.25 mm × 0.25 μm) with the following temperature program: initial temperature 40 °C (hold 3 min), ramped to 230 °C at 4 °C/min (hold 10 min). The helium carrier gas was maintained at a constant flow rate of 1.2 mL/min.

Mass spectrometric detection employed electron ionization (70 eV) with an ion source temperature of 230 °C. Compounds were identified by matching mass spectra against the NIST 2017 library and confirmed using linear retention indices. Semi-quantitative analysis was performed by normalizing peak areas to the internal standard.

### 2.7 Microbial community analysis

Total genomic DNA was extracted using the FastDNA SPIN Kit (MP Biomedicals, Santa Ana, CA, United States) according to the manufacturer’s instructions. DNA integrity was assessed by electrophoresis on 0.8% (w/v) agarose gel and concentration and purity were measured with a NanoDropND-1000 spectrophotometer (Thermo Scientific, Waltham, MA, United States).

PCR Amplification: Bacterial 16S rRNA V3–V4 region using primers 338F (5′-ACTCCTACGGGAGGCAGCAG-3′) and 806R (5′- GGACTACHVGGGTWTCTAAT-3′); Fungal ITS1 region using primers ITS5 (5′-GGAAGTAAAAGTCGTAACAAGG-3′) and ITS1 (5′-TCCGTAGGTGAACCTGCGG-3′). Prepared reaction mixtures were subjected to PCR under the following conditions: initial denaturation at 98 °C for 5 min, followed by amplification cycles. Each cycle consisted of denaturation at 98 °C for 30 s, annealing at 52 °C for 30 s (for fungi, 55 °C for 45 s), and extension at 72 °C for 45 s. A total of 25 cycles (30 cycles for fungi) were performed. Finally, the samples were subjected to a final extension at 72 °C for 5 min and then held at 12 °C. Amplicons were visualized on 2% agarose gels, and target bands were excised and purified using the AxyPrep DNA Gel Extraction Kit (Axygen Biosciences, Union City, CA, United States). Equimolar amplicon pools were sequenced on an Illumina MiSeq platform (BGI Genomics, Shenzhen, China) with 2 × 300 bp paired-end chemistry. Sequence processing followed the DADA2 pipeline, including quality filtering (maxN = 0, truncQ = 2), error rate modeling, dereplication, chimera removal, and amplicon sequence variant (ASV) inference. Taxonomic assignment was performed using the SILVA 138 database for 16S rRNA sequences and the UNITE 8.3 database for ITS sequences at 97% similarity.

### 2.8 Statistical analysis

All experiments were performed in biological triplicate. Data are expressed as mean ± standard deviation (SD). Significant differences (*p* < 0.05) were evaluated by one-way analysis of variance (ANOVA) followed by Duncan’s multiple range test using SPSS 25.0 (IBM Corp., Armonk, NY, United States).

Multivariate analysis was conducted using partial least squares-discriminant analysis (PLS-DA) in SIMCA-P + 14.1 (Umetrics, Umea, Sweden) with unit variance scaling. Data visualization comprised: Column and line charts in OriginPro 2022 (OriginLab Corp., Northampton, MA, United States); Co-occurrence networks constructed in Gephi 0.10.1 (Fruchterman-Reingold layout); and model validation through seven-fold cross-validation and 200 iterations permutation testing.

To prioritize robust and biologically interpretable associations for the main text, we focused on genus–metabolite correlations meeting a conservative threshold of |*ρ*| ≥ 0.60 with a false discovery rate (FDR)-adjusted q-value < 0.05. The full correlation matrix, including all tested edges with their respective ρ and q-values, is available in Supplementary Table S4.

## 3 Results and discussion

### 3.1 Variation in physicochemical parameters during fermentation

**Figure 1:**
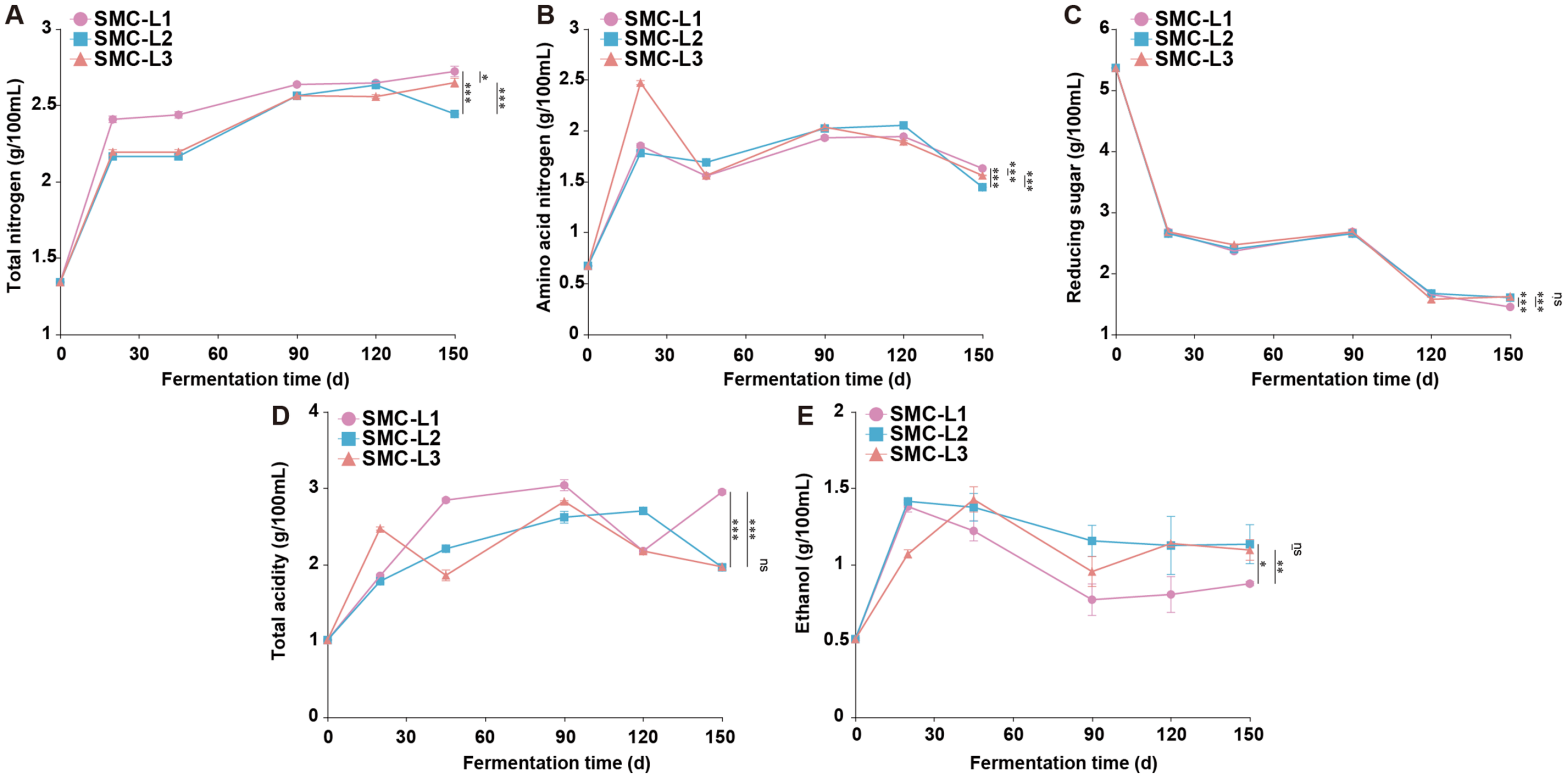
Changes in physical and chemical indicators during the fermentation process of reduced-salt sauce. *p < 0.05, **p < 0.01, ***p < 0.001. **(A)** Total nitrogen. **(B)** Amino acid nitrogen. **(C)** Reducing sugar. **(D)** Total acidity. **(E)** Ethanol.

**Figure 2:**
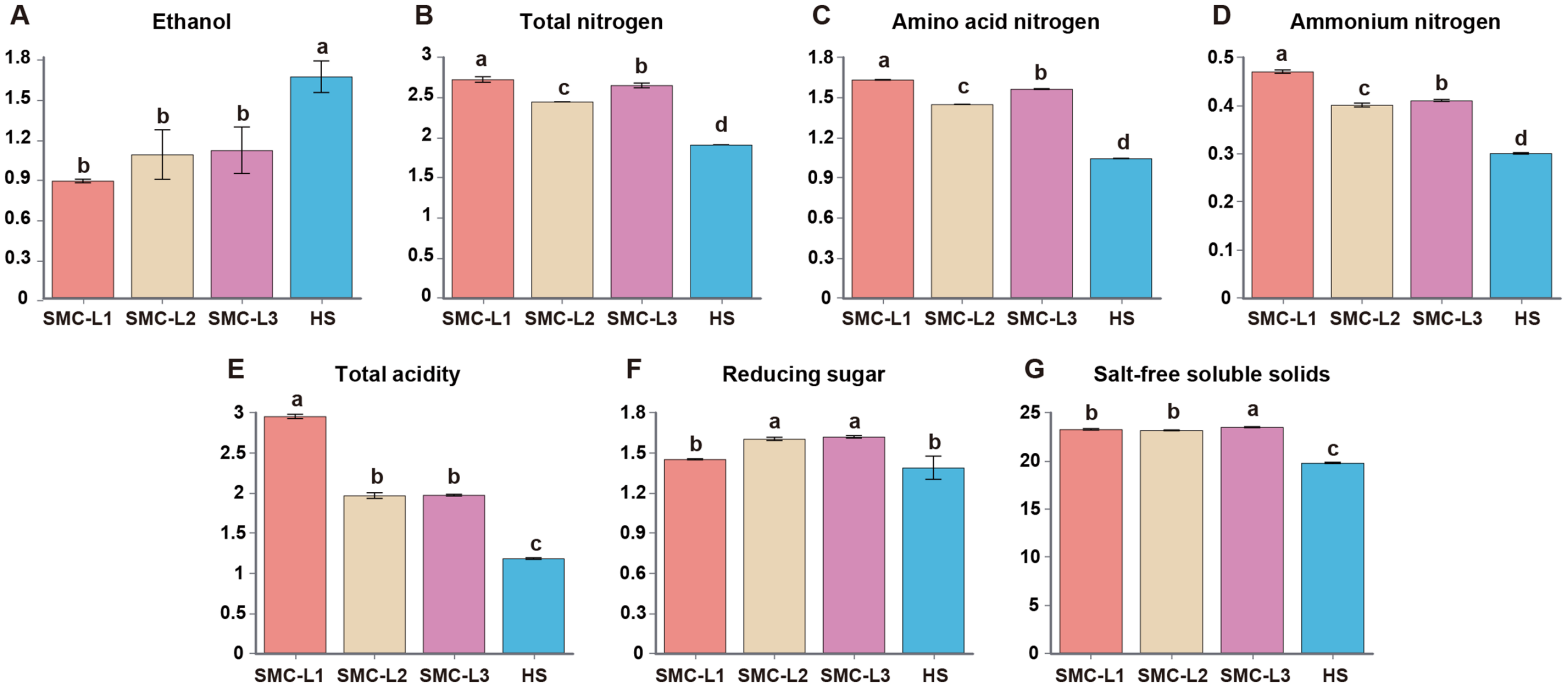
Physical and chemical indicators were measured after 5 months of fermentation of the sauce (Significant differences were determined using Duncan’s test). **(A)** Ethanol. **(B)** Total nitrogen. **(C)** Amino acid nitrogen. **(D)** Ammonium nitrogen. **(E)** Total acidity. **(F)** Reducing sugar. **(G)** Salt-free soluble solids.

The physicochemical trajectories revealed distinct functional outcomes (Figure 1). Fermentation with *L. plantarum* (SMC-L3) induced pronounced acidification (total acidity 2.47 g/100 mL) within 20 days, which concurrently suppressed and delayed ethanol synthesis. The TA in the SMC-L1 and SMC-L3 groups peaked earlier (on day 90) than in the SMC-L2 group (day 120). Meanwhile, a decrease in reducing sugars from 53.60 g/L to 14.50–16.20 g/L indicated active microbial consumption. After 5 months, all inoculated SynMC groups met the criteria for premium-grade soy sauce (GB/T 18186–2000) (Figure 2). The evaluation of pre-defined endpoints—including total nitrogen (TN), amino acid nitrogen (AAN), and key metabolites—established that the SMC-L1 consortium consistently outperformed other SynMCs and, crucially, matched or exceeded the high-salt control in metrics critical to sensory quality. Specifically, compared to the high-salt control, SMC-L1 fermentation resulted in a 40.8% increase in TN (2.69 g/100 mL) and a 56.7% increase in AAN (1.63 g/100 mL). Additionally, TA and salt-free soluble solids were also significantly elevated (*p* < 0.05), suggesting an enhanced overall profile. The superior performance of SMC-L1 demonstrates the success of a defined SynMC for reduced-salt soy sauce fermentation. This outcome validates our screening strategy, which successfully identified *T. halophilus* T10 as the optimal LAB module for achieving functional synergy with a stable yeast consortium under 13% NaCl stress. Moreover, the temporal partitioning of community assembly observed in Figure 1 is consistent with reduced inter-guild conflict, potentially explaining the coordinated metabolite production. The declines in AAN and ethanol in later stages are likely due to their ongoing microbial conversion into esters and organic acids, respectively (Chen et al., 2024). While this pattern is compelling, we note that the absence of high-frequency kinetic data means the mitigation of antagonism, though consistent with our model, is not directly proven here and warrants future investigation.

### 3.2 Differential analysis of non-volatile compounds in sauce mash

**Figure 3:**
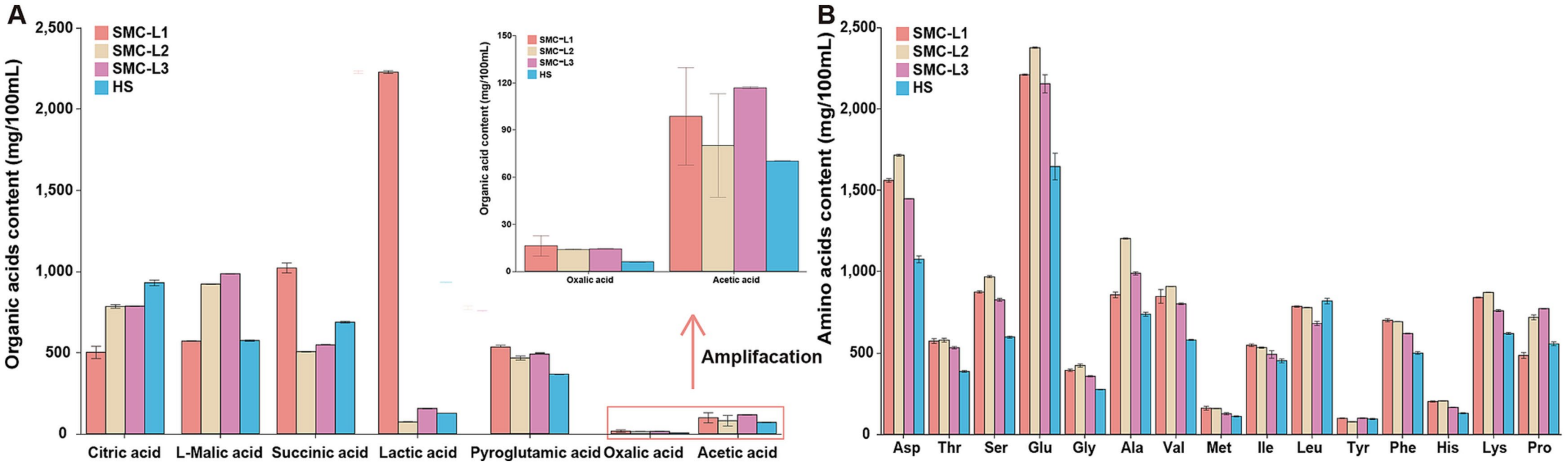
The content of organic acids **(A)** and free amino acids **(B)** in the fermented mash at the end of fermentation.

**Figure 4:**
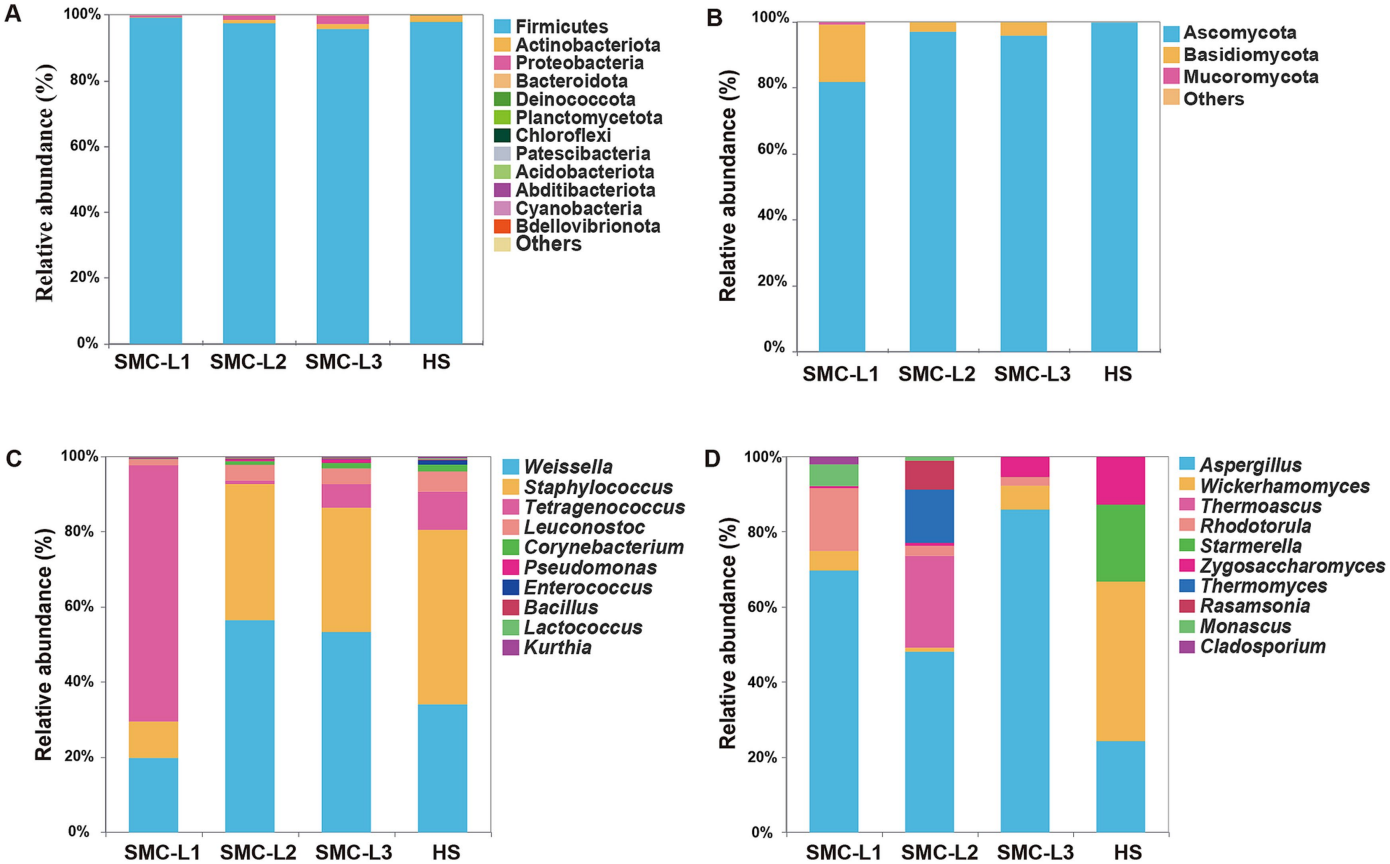
Relative abundance of bacterial **(A)** and fungal **(B)** community at the phylum level and relative abundance of bacterial **(C)** and fungal **(D)** community at the genus level.

The reduced-salt fermentation markedly altered the organic acid (OA) and free amino acid (FAA) composition of moromi, thereby influencing its flavor profile (Figure 3A). Upon completion of fermentation, samples SMC-L2 and SMC-L3 exhibited similar total OA concentration (3640.51 mg/100 mL and 3869.95 mg/100 mL, respectively), whereas SMC-L1 contained significantly more (5203.57 mg/100 mL), representing a 37.68% increase compared to sample HS. Succinic acid, L-malic acid, and citric acid were the predominant OAs. Moreover, SMC-L1 showed the most pronounced intergroup changes: lactic acid showed a 17.68-fold increase compared to HS, succinic acid was 48.58% higher, and pyroglutamic acid increased by 52.9%. These shifts may enhance umami characteristics (Uddin et al., 2024) and reflect the distinct bacterial community in SMC-L1 (Figure 4).

Sixteen FAAs were detected, with total concentrations ranging from 87.97 to 125.14 mg/mL (Figure 3B). FAA composition directly influences the moromi taste and quality (Wang et al., 2021; Chen et al., 2024). Sample HS contained 87.97 mg/mL of total FAA. In comparison, all reduced-salt sauce groups had significantly elevated total FAA levels compared to the HS group. The increases were in the order of SMC-L2 (42.26%) > SMC-L1 (30.53%) > SMC-L3 (26.05%) (*p* < 0.05). The FAAs were categorized by taste: six sweet FAAs (Ala, Gly, Ser, Thr, Pro, Lys) ranged from 31.68 to 47.59 mg/mL (36.13–39.13% of total FAA), two umami FAAs (Asp., Glu) ranged from 27.20 to 40.92 mg/mL (31.76–33.90% of total FAA), and eight bitter FAAs (Arg, His, Ile, Leu, Met, Phe, Tyr, Val) ranged from 26.76 to 33.48 mg/mL (27.44–31.25%). All FAAs except Leucine increased (15 of 16) in the reduced-salt group. In SMC-L1, umami, sweet, and bitter FAAs comprised 38.61, 26.84, and 24.60% of total FAAs, respectively. These findings indicate that synthetic microbiota promote FAA accumulation via metabolic regulation, particularly enhancing umami FAAs. Overall, SMC-L1 increased OA and FAA content, especially umami components, through its unique bacterial community, suggesting a key mechanism for flavor optimization (Figure 4).

### 3.3 Differences in volatile components

**Figure 5:**
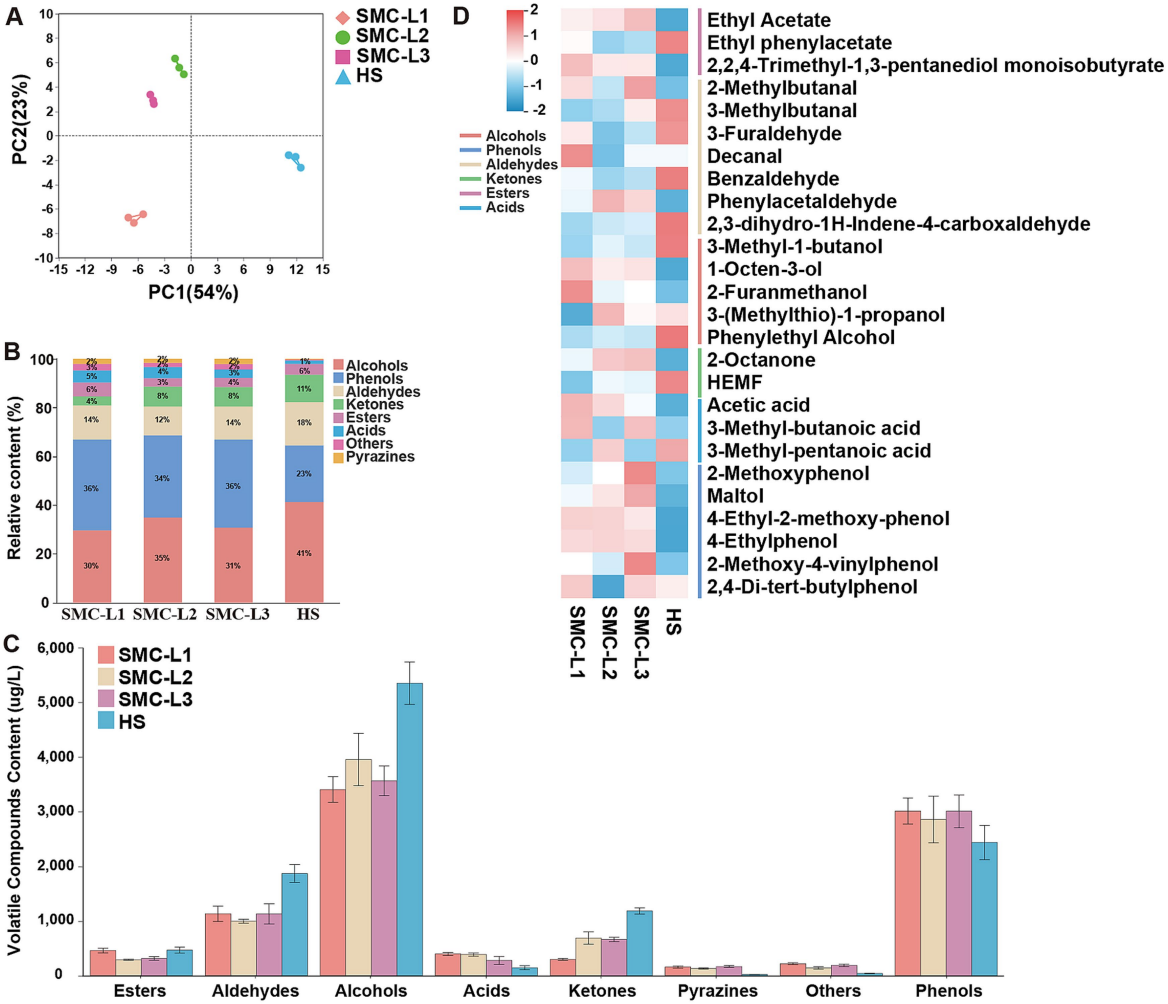
The volatile compounds analysis for moromi. **(A)** The PCA analysis of volatile compounds in moromi. **(B)** The proportions of volatile compounds in moromi. **(C)** Concentrations of volatile compounds. **(D)** Heatmap of main volatile compounds.

Reduced-salt fermentation markedly altered the volatile organic compounds (VOC) composition of soy sauce, thereby modulating its flavor profile. At the fermentation endpoint, 100 VOCs were identified, including 12 alcohols, 25 esters, 19 aldehydes, 10 ketones, 12 acids, 7 phenols, 9 pyrazines, and 6 other compounds (Supplementary Table S1). Thirty-three VOCs were common across all samples, consistent with Zhang et al. (2024a). Although total VOC abundance did not differ significantly between reduced-salt and control (HS) groups, their compositional profiles diverged. Principal Component Analysis (PCA) (Figure 5A) positioned SMC-L1 (functional microbiota-enhanced) in quadrant III, SMC-L2 and SMC-L3 in quadrant II, and HS in quadrant IV. The stacked bar chart (Figure 5B) indicated that alcohols, phenols, and aldehydes were the dominant contributors. Notably, the reduced-salt groups exhibited higher phenol content (33.78–37.20%) than HS (23.17%), whereas alcohols predominated in HS (41.35%). Concurrently, the contents of aldehydes and ketones decreased by 39.26–46.59 and 41.72–74.65%, respectively, (Figure 5C). In contrast, the contents of acids and pyrazines increased by 91.22–171.76 and 17.20–23.52%, respectively. Pyrazine levels in enhanced groups reached 132.40–169.85 μg/mL, significantly enriching baking aroma (Zheng et al., 2024). Moreover, Figure 5D presents a heatmap constructed by selecting key flavor compounds with VIP scores greater than 1 and those previously reported by Zhao et al. (2020) as having notably high odor activity values (OAVs). Subsequent analysis revealed that functional fermentation increased ethyl acetate (fruity aroma), furfuryl alcohol, and 1-octen-3-ol (mushroom aroma) (Wang et al., 2024; Wang et al., 2024), along with phenylacetaldehyde in SMC-L2 and decanal in SMC-L1, effectively suppressing oxidative off-odors from fatty acid-derived aldehydes (Zhou et al., 2024). Altered phenethyl alcohol content likely reflects reduced yeast relative abundance (RA) and metabolic pathway shifts (Figure 4). Notably, 5-hydroxymethyl-2-furfural (HEMF) content decreased in enhanced reduced-salt mash, warranting further mechanistic investigation. Overall, this strategy optimized flavor by increasing pyrazine-to-ester ratios and decreasing aldehyde-to-alcohol ratios. Collectively, SynMC fortification under reduced salt produced coordinated shifts in esters, phenols, higher alcohols, and succinate. These are changes in sensory-relevant chemical pools; we do not infer perceptual outcomes because no sensory testing was conducted.

### 3.4 Analysis of microbial community differences

To assess the effect of SynMC enhancement on reduced-salt moromi microbiota, we first evaluated sequencing data quality and depth. For bacteria, effective sequences numbered from 76,381-87,483, of which 72,309–78,840 (82.76–94.67%) passed quality filters. For fungi, effective sequences numbered 90,760-126,024, with 84,131–99,663 (80.08–92.70%) high-quality reads (Supplementary Table S2). Coverage curves confirmed sufficient depth for representative community profiling. Alpha diversity metrics (Chao1/observed species indices) are summarized in Supplementary Table S3. Richness and diversity of bacterial microbiota significantly exceeded fungal values (*p* < 0.05). Compared to the control (HS), SMC-L3 exhibited a 124.24% increase in bacterial richness, whereas fungal richness in SMC-L1 and SMC-L3 decreased slightly. Hierarchical clustering (Supplementary Figure S1) showed that bacterial communities clustered separately from SMC-L2 and HS, while SMC-L1’s fungal community clustered closer to SMC-L3 but diverged most from HS.

Phylum-level analysis (Figures 4A,B) identified 18 bacterial phyla dominated by Firmicutes (95.77–99.21% RA; SMC-L1 > SMC-L2 > HS > SMC-L3) and six fungal phyla dominated by Ascomycota (81.89–99.97% RA). At the genus level, the top 10 bacterial genera accounted for 97.91–99.48% of sequences, chiefly *Weissella* (19.69–55.79%), *Staphylococcus* (9.62–46.28%), and *Tetragenococcus* (0.91–67.86%), consistent with Jiang et al. (2023). By fermentation endpoint, *Tetragenococcus* RA in SMC-L1 reached 67.86%, correlating with its robust acid production and SMC-L1’s highest total acidity (Figure 2). Conversely, *Weissella* dominated SMC-L2 and SMC-L3, while *Staphylococcus* dominated HS. *Weissella*, *Staphylococcus*, *Leuconostoc*, and *Bacillus* are established functional taxa that degrade macromolecules and synthesize flavor compounds (Liu et al., 2021; Zhang et al., 2024a).

SynMC enhancement also reshaped fungal genera (Figure 4D), with *Aspergillus* increasing from 24.23% RA in HS to 67.28–83.60% in enhanced groups. Salt reduction alleviates NaCl-induced stress on *A. oryzae*, as demonstrated by a reported 97% drop in its protease activity at 18% NaCl (Su et al., 2005), thereby improving its colonization, central carbon metabolism, and symbiosis with lactic acid bacteria (Kuang et al., 2022). Consequently, *Aspergillus*’ dual starch/cellulose-degrading functions were enhanced (Xiang et al., 2024). Meanwhile, yeast RA declined from 75.58 to 12.18–21.70%, further modifying fungal structure. These microbiota shifts explain the improved physicochemical parameters and elevated amino acid content in reduced-salt groups (Figures 2, 3B). In summary, Syn enhancement significantly restructured sauce mash microbiota, enhancing bacterial richness, increasing fungal complexity, and selectively promoting *A. oryzae* via salt stress alleviation.

### 3.5 Correlation analysis between microbial communities and metabolites

**Figure 6:**
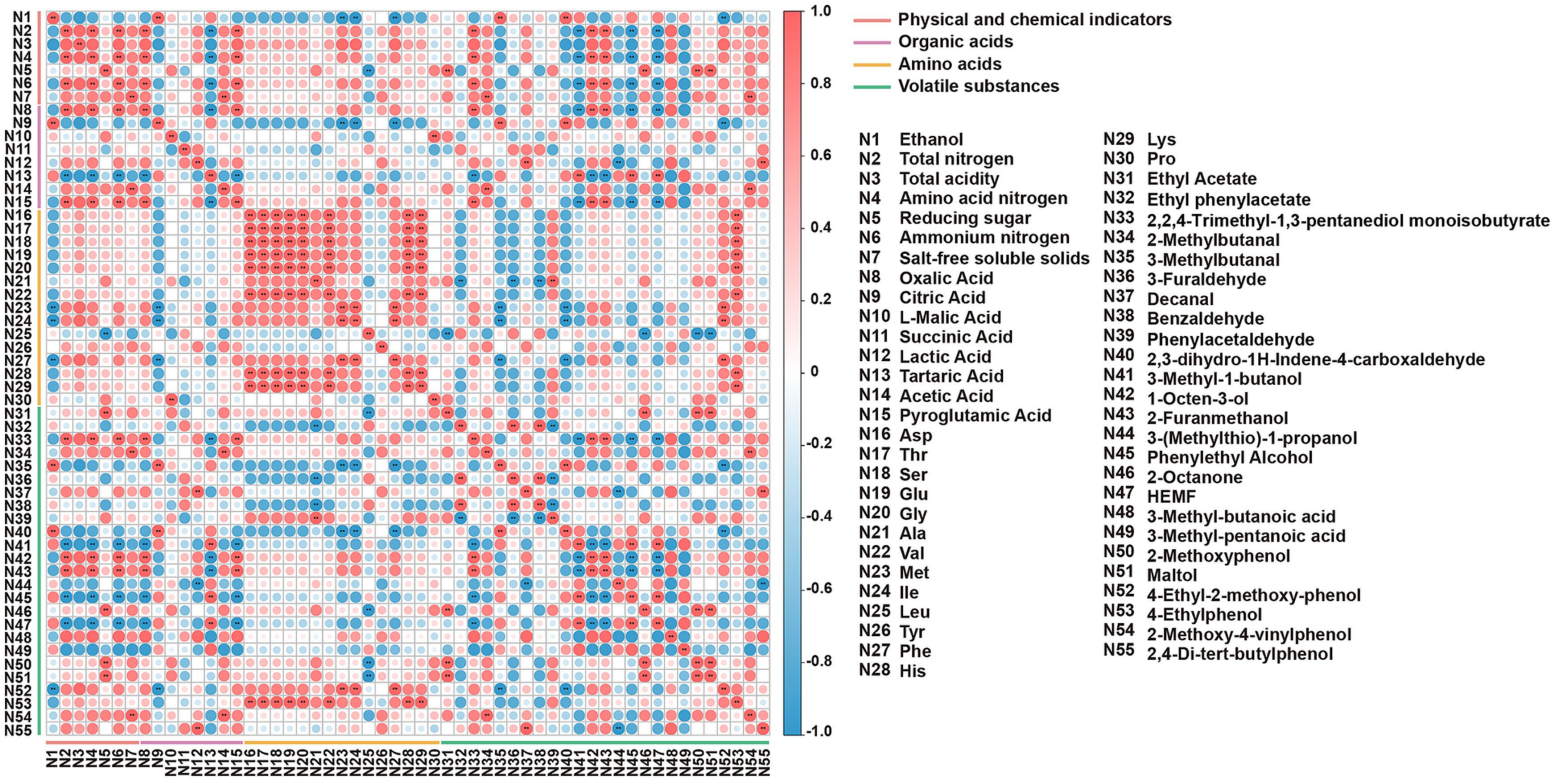
The heatmap of correlations among differential metabolites. *p < 0.05, **p < 0.01, ***p < 0.001.

**Figure 7:**
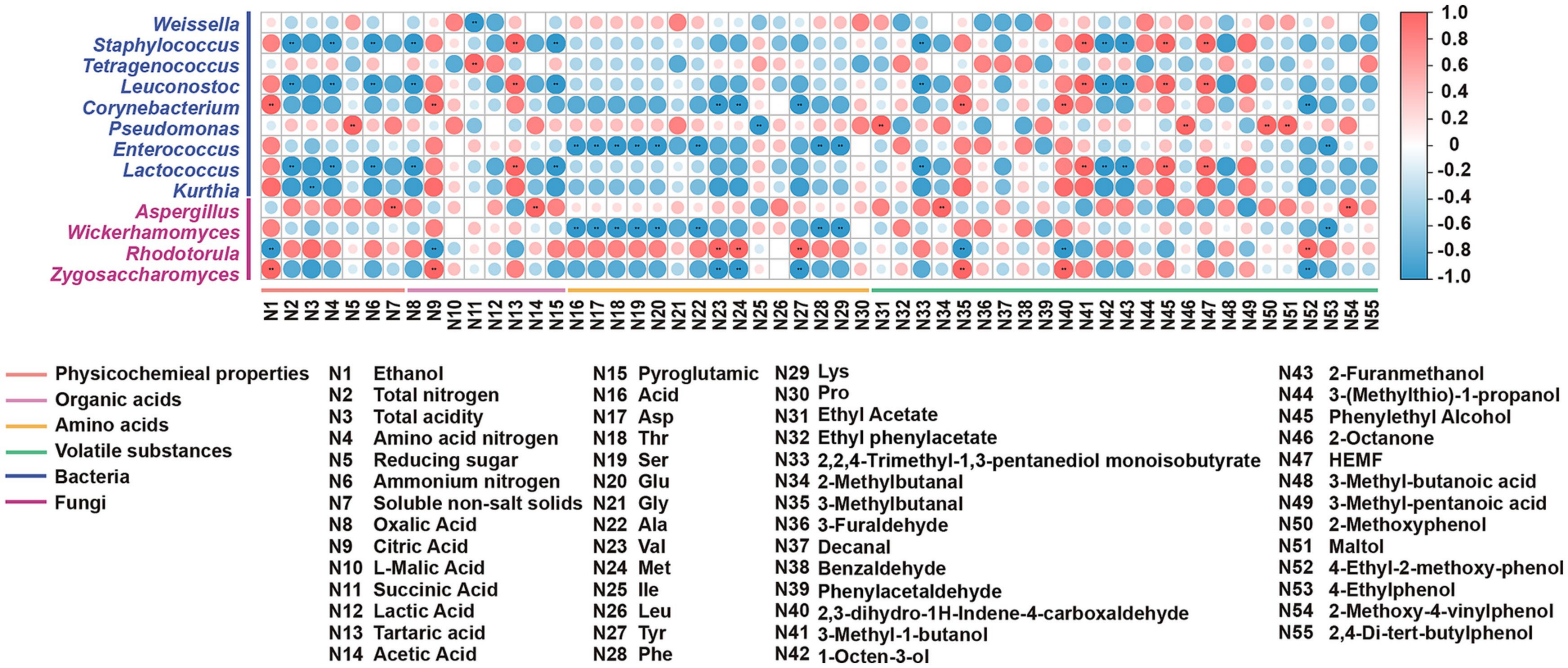
The heatmap of correlations between dominant microbes and differential metabolites. *p < 0.05, **p < 0.01, ***p < 0.001.

Spearman’s correlation and network analysis (Figures 6, 7) uncovered structured relationships between the microbiome and metabolites in reduced-salt fermentation. This allowed us to move beyond mere associations and propose mechanistic links between community structure and functional outcomes. After applying conservative statistical filtering (|*ρ*| ≥ 0.6; FDR-adjusted q < 0.05), we identified three reproducible ecology-to-chemistry axes that explain the majority of coordinated metabolic shifts (Figures 6, 7). First, *Tetragenococcus* correlated positively with succinate, which is consistent with LAB anaplerotic routing under saline, microaerobic conditions. Second, the net positive correlation between *Tetragenococcus* and short-chain esters is likely an indirect effect mediated by increased lactate/acetate pools and lowered pH, conditions that favor both yeast alcohol-acyltransferase activity and chemical esterification. Third, *Aspergillus is* also correlated positively with FAA. This aligns with the robust protease/peptidase activity of *Aspergillus*, which elevates levels of glutamic acid, aspartic acid, and branched-chain amino acids. These subsequently feed the yeast Ehrlich pathway for the production of higher alcohols and their downstream esters.

These targeted microbial-metabolite axes provide a functionally anchored explanation linking community restructuring to the observed metabolite outcomes. The enrichment of *Tetragenococcus* expands the succinate pool via the reductive branch of the TCA cycle and, by elevating lactate/acetate availability and acidifying the environment, indirectly promotes the formation of short-chain esters, resulting in a flavor profile characterized by increased fruity/buttery esters and succinate, and reduced higher alcohols. In parallel, the increased abundance of *Aspergillus* enhances proteolysis, elevating FAA levels that supply precursors for the yeast Ehrlich pathway. This yields higher alcohols, which subsequently esterify with the LAB-derived acid pool. Together, these parsimonious, function-driven relationships explain the coordinated increases in succinate, FAAs, higher alcohols, and esters observed under reduced-salt with SMC-L1 fortification.

The observed metabolic changes are best interpreted as chemical indicators with established sensory relevance. It is crucial to note that these correlations constitute a powerful mechanistic hypothesis linking community restructuring to chemistry, not a direct demonstration of perceptual effects, which requires future sensory evaluation.

Thus, our interpretation of reduced inter-guild antagonism relies on integrative, but ultimately cross-sectional, evidence—namely, final community composition and metabolite profiles—rather than direct kinetic validation. Therefore, mitigation of antagonism should be considered a plausible mechanism consistent with our results, not a definitively demonstrated outcome.

### 3.6 Metabolic pathway analysis of key substances in soy sauce fermentation

**Figure 8:**
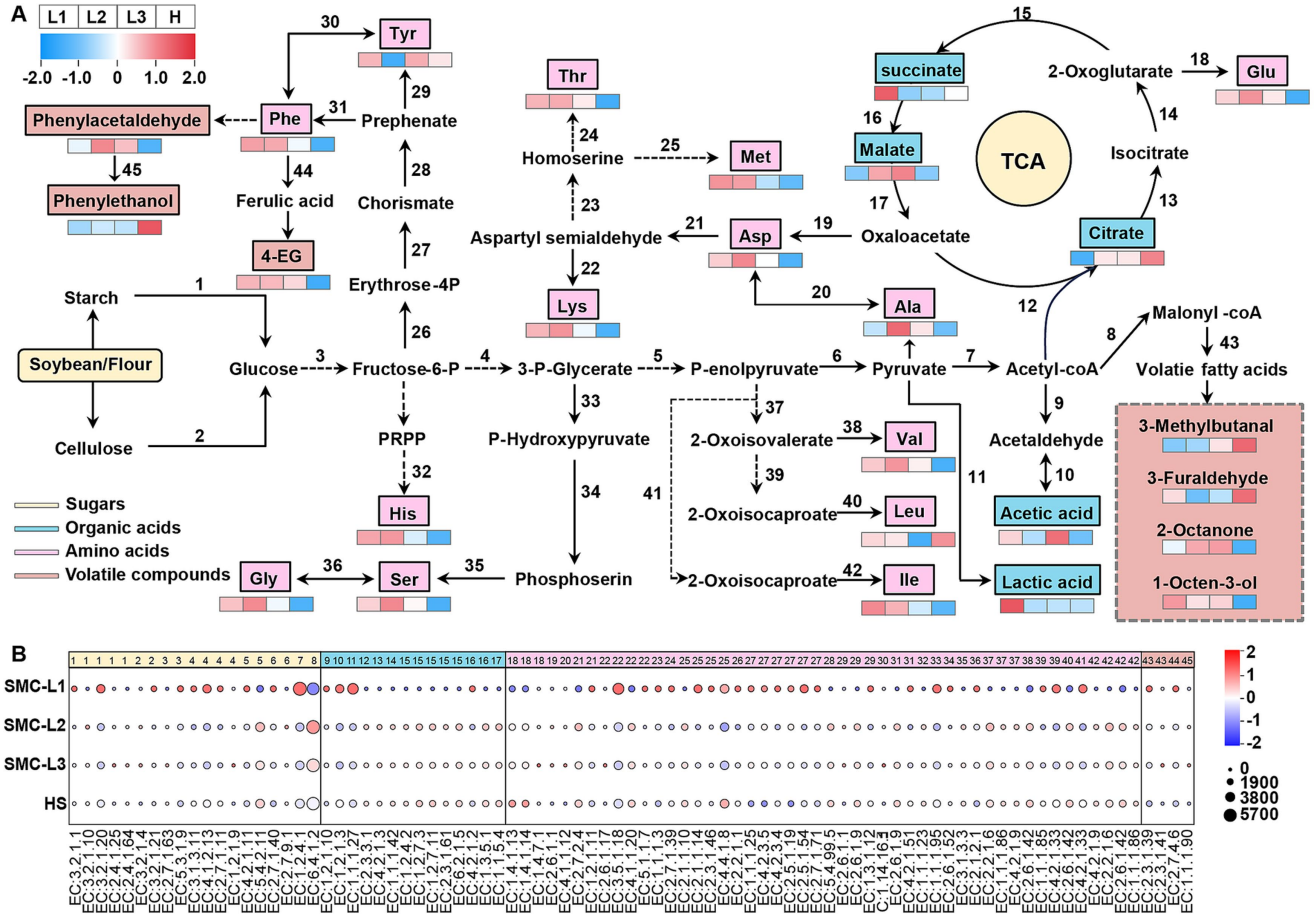
Predicted metabolic networks related to carbohydrate metabolism, amino acid metabolism, and the formation of different flavors in soy sauce **(A)** and TPM abundance of annotated enzymes in each type of soy sauce in metabolomics **(B)**.

Based on our comparative reconstruction of the KEGG and MetaCyc databases, we delineated major metabolic pathways active during fermentation process, with emphasis on carbohydrate and amino acid metabolism (Figure 8A). A corresponding heatmap (Figure 8B) visualizes differential enzyme abundance (TPM), where dot size corresponds to enzyme levels and color reflects normalized intergroup variation. Of all conditions, SMC-L1 demonstrated the most substantial shifts in enzymatic profiles.

Carbohydrates, key substrates for microbial metabolism, were degraded via starch and cellulose hydrolysis, entering glycolysis primarily through the EMP pathway. The reduced-salt group (L) showed increased abundance of starch- and cellulose-degrading enzymes compared to the high-salt control (HS) (Figure 2), aligning with its higher glucose content. This enhancement is likely due to the enrichment of *Aspergillus* in group L (Figure 4D), which secretes potent carbohydrate-active enzymes. Subsequent glycolysis generated pyruvate and acetyl-CoA, which entered the TCA cycle. In SMC-L1, upregulation of pyruvate kinase (EC 2.7.1.40) and pyruvate dehydrogenase (EC 1.2.4.1) supplied critical precursors for organic acid and alcohol biosynthesis. Dominance of *T. halophilus* T10 in SMC-L1 (Figure 4C) further promoted succinate accumulation via enhanced TCA flux.

Late in fermentation, oxygen depletion stimulated the proliferation of *T. halophilus* T10 (Figure 4D), shifting pyruvate utilization toward lactate synthesis. Accordingly, SMC-L1 displayed increased lactate/acetate pathway activity accompanied by reduced TCA cycle enzyme expression. Total acidity rose significantly after four to 5 months (Figure 1). In contrast, organic acid-metabolizing enzyme levels in SMC-L2 and SMC-L3 remained comparable to the high-salt control.

FAAs critically influence soy sauce flavor. Among 48 FAA-metabolizing enzymes analyzed (Figure 8B), activity followed the order: SMC-L1 > SMC-L2 ≈ SMC-L3 > HS, indicating that reduced-salt conditions promote amino acid metabolism, potentially via synergistic interactions between acid-tolerant microbes and *Aspergillus*. However, umami-associated amino acids (e.g., glutamate, aspartate) did not consistently correlate with expression of key biosynthetic enzymes such as glutamate synthase (EC 1.4.1.13/14) and aspartate transaminase (EC 2.6.1.1), suggesting that their accumulation occurs mainly during early fermentation.

Additionally, phenylalanine-metabolizing enzyme activity increased markedly under reduced salt, particularly in SMC-L1, supporting elevated levels of phenolic volatiles like phenylacetaldehyde and 4-ethylguaiacol (see section 3.3). Conservative statistical filtering (|*ρ*| ≥ 0.60; FDR-adjusted q < 0.05) identified three high-confidence, reproducible ecology–metabolite associations: (i) *Tetragenococcus* ↔ succinate, (ii) *Tetragenococcus* ↔ short-chain esters (net positive), and (iii) *Aspergillus* ↔ FAAs. These linkages align with observed changes in organic acids, FAAs, alcohols, and esters across reduced-salt batches (see full correlation set in Supplementary Table S4). Two functional axes underpin the metabolic restructuring under SMC supplementation and salt reduction: First, the positive *Tetragenococcus*–succinate correlation reflects the capacity of halophilic LAB to redirect phosphoenolpyruvate via oxaloacetate into the reductive TCA branch (through malate and fumarate) under saline and microaerobic conditions. The association between *Tetragenococcus* and short-chain esters (e.g., ethyl acetate, ethyl lactate) is likely indirect: by elevating lactate/acetate availability and lowering pH, both Yao et al. (2019) and Cui et al. (2014) discuss the role of LAB and yeast coculture in ester synthesis, supporting. Second, the *Aspergillus*-FAA correlation aligns with its strong secretory capacity for proteases, peptidases, and amylases, which hydrolyze peptides and polysaccharides to release FAAs (e.g., glutamate, aspartate, and branched-chain amino acids). These FAAs subsequently feed the yeast Ehrlich pathway, yielding higher alcohols that esterify with the acid pool derived from LAB. This mechanistic model links microbial community structure to the enhanced production of key flavor and aroma compounds. In summary, SMC-driven fermentation under salt reduction coordinated the levels of esters, phenols, higher alcohols, and succinate. These shifts indicate profound restructuring of sensory-relevant chemistry. However, as no formal sensory analysis was conducted, we restrict our conclusions to chemical and microbial changes, avoiding extrapolation to perceptual outcomes.

## 4 Conclusion

This study demonstrates that a defined SynMC can sustain fermentation stability and optimize flavor-relative chemistry under reduced-salt conditions. The SMC-L1 consortium produced the most pronounced enzymatic and metabolic shifts, with enhanced carbohydrate degradation linked to elevated glucose flux through glycolysis and precursors for organic acid and alcohol biosynthesis. Three robust ecology-to-chemistry axes were consistently observed: (i) a positive associated between *Tetragenococcus* and succinate consistent with routing through the reductive TCA branch; (ii) a net positive association between *Tetragenococcus* and short-chain esters, plausibly mediated by lactate/acetate accumulation and pH reduction that favor esterification; and (iii) a positive association between *Aspergillus* and FAAs, arising from protease/peptidase activity that supplies substrates for yeast Ehrlich pathways. Together these interactions coordinated increases in esters, phenolic derivatives, higher alcohols, and succinate. Our conclusions are restricted to chemical and microbial endpoints; future work will scale the process and verify sensory outcomes with formal evaluation.

## Data Availability

The Illumina paired-end 16S rRNA data presented in the study are deposited in the Genome Sequence Archive repository, this data can be found here: https://ngdc.cncb.ac.cn/gsa/browse/CRA032131.
