# Supplementary material for: Synthetic microbial community SMC-L1 optimizes flavor chemistry in reduced salt soy sauce via targeted metabolic reprogramming
Source: Front Microbiol. 2025 Nov 12;16:1701479. doi: 10.3389/fmicb.2025.1701479 (PMC12657017; doi:10.3389/fmicb.2025.1701479)
Supplement: Supplementary file 1 [file Table_1.docx]

Supplementary Material

# Supplementary Figures and Tables

## Supplementary Figures


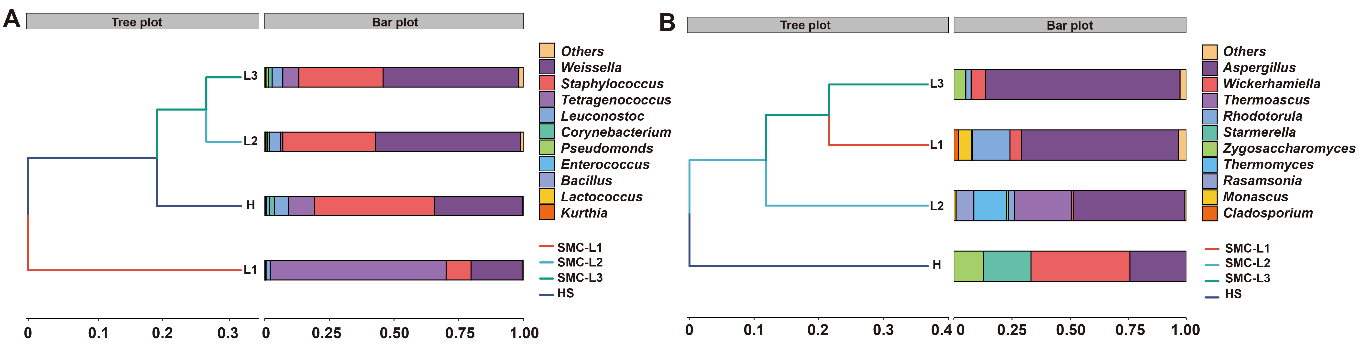


Figure S1 Hierarchical clustering of bacterial (A) and fungal (B) community at the genus leve

## Supplementary Tables

**Table S1** Qualitative and quantitative results of volatile compounds of different groups of sauce by HS-SPME/GC-MS

|  | Number | Compound | Concentration of volatile compounds (µg/L) | | | |
| --- | --- | --- | --- | --- | --- | --- |
|  |  |  | SMC-L1  (*T. halophilus* T10) | SMC-L2  (*T. halophilus* CGMCC3792) | SMC-L3  (*L. plantarum*1.08) | HS  (comparison) |
| Esters | 1 | 1-Propen-2-ol, acetate | 34.33±3.89^a^ | 30.47±2.23^a^ | 34.38±4.04^a^ | 0^b^ |
|  | 2 | Ethyl Acetate | 103.33±10.15^a^ | 106.27±9.25^a^ | 113.65±18.12^a^ | 77.09±7.17^b^ |
|  | 3 | 1-Butanol, 3-methyl-, acetate | 5.46±0.08^a^ | 0^b^ | 0^b^ | 0^b^ |
|  | 4 | Hexanoic acid, ethyl ester | 4.35±0.82^a^ | 0^b^ | 0^b^ | 0^b^ |
|  | 5 | Propanoic acid, 2-hydroxy-, ethyl ester, (L)- | 65.38±9.06^a^ | 0^b^ | 0^b^ | 0^b^ |
|  | 6 | S-Methyl 3-methylbutanethioate | 14.25±2.96^a^ | 0^b^ | 0^b^ | 0^b^ |
|  | 7 | Pentanoic acid, 2-hydroxy-4-methyl-, ethyl ester | 3.91±0.31^a^ | 0^b^ | 0^b^ | 0^b^ |
|  | 8 | 9-Octadecenoic acid (Z)-, phenylmethyl ester | 6.32±1.42^ab^ | 5.2±0.46^b^ | 7.12±0.01^a^ | 0^c^ |
|  | 9 | Butanedioic acid, diethyl ester | 17.93±5.03^a^ | 0^c^ | 2.03±0.13^bc^ | 6.83±0.88^b^ |
|  | 10 | Cyclohexane, (methylthio)- | 8.42±1.63^c^ | 23.46±1.77^bc^ | 29.73±3.5^b^ | 153.44±15.71^a^ |
|  | 11 | Benzeneacetic acid, ethyl ester | 28.58±1.55^b^ | 17.71±2.59^c^ | 19.99±0.9^c^ | 44.08±3.11^a^ |
|  | 12 | Acetic acid, 2-phenylethyl ester | 17.05±0.86^a^ | 0^d^ | 10.23±0.21^c^ | 12.76±1.03^b^ |
|  | 13 | Undecanoic acid, 3-hydroxy-, methyl ester | 18.75±3.21^a^ | 10.76±0.57^b^ | 0^c^ | 0^c^ |
|  | 14 | Propanoic acid, 2-methyl-, 3-hydroxy-2,2,4-trimethylpentyl ester | 73.92±5.89^a^ | 56.62±6.48^b^ | 56.84±7.13^b^ | 0^c^ |
|  | 15 | Dehydromevalonic lactone | 6.88±0.7^a^ | 6.98±1.21^a^ | 7.92±1.17^a^ | 0^b^ |
|  | 16 | γ-Dodecalactone | 18.64±1.88^a^ | 17.82±0.32^a^ | 0^b^ | 20.18±3.99^a^ |
|  | 17 | Phthalic acid, butyl undecyl ester | 12.18±0.43^a^ | 10.98±6.56^a^ | 16.83±3.55^a^ | 0^b^ |
|  | 18 | Phthalic acid, butyl nonyl ester | 16.06±1.9^a^ | 0^b^ | 0^b^ | 0^b^ |
|  | 19 | Hexadecanoic acid, ethyl ester | 7.52±0.11^b^ | 6.91±0.21^b^ | 0^c^ | 17.36±3.67^a^ |
|  | 20 | Propanoic acid, ethenyl ester | 0^c^ | 0^c^ | 5.87±0.12^b^ | 12±0.92^a^ |
|  | 21 | Octaethylene glycol monododecyl ether | 0^b^ | 0^b^ | 11.97±6.67^b^ | 22.23±4.62^a^ |
|  | 22 | Benzoic acid, ethyl ester | 0^b^ | 0^b^ | 0^b^ | 29.38±1.51^a^ |
|  | 23 | Cyclohexanol, 3,3,5-trimethyl-, acetate, cis- | 0^b^ | 0^b^ | 0^b^ | 29.52±6.69^a^ |
|  | 24 | Undec-10-ynoic acid, nonyl ester | 0^b^ | 0^b^ | 0^b^ | 32.6±9.5^a^ |
|  | 25 | [1,1'-Bicyclopropyl]-2-octanoic acid, 2'-hexyl-, methyl ester | 0^b^ | 0^b^ | 0^b^ | 10.79±5.47^a^ |
| Ketones | 26 | 2-Butanone | 16.99±1.81^a^ | 10.2±0.75^b^ | 12.02±1.27^b^ | 7.12±0.23^c^ |
|  | 27 | 2-Octanone | 179.85±6.72^b^ | 207.56±8.59^a^ | 211.01±3.65^a^ | 137.74±11.31^c^ |
|  | 28 | 5-Hepten-2-one, 6-methyl- | 5.35±0.32^a^ | 0^c^ | 4.73±0.04^b^ | 0^c^ |
|  | 29 | 2-Methyl-3-methoxy-4H-pyran-4-one | 12.81±0.71^a^ | 13.93±2.42^a^ | 15.27±0.49^a^ | 0^b^ |
|  | 30 | 2-Cyclopenten-1-one, 3-ethyl-2-hydroxy- | 20.16±1.02^a^ | 0^b^ | 0^b^ | 0^b^ |
|  | 31 | 5-Ethyl-4-hydroxy-2-methyl-3(2H)-furanone, acetate | 65.74±25.31^c^ | 429.55±105.46^b^ | 398.85±39.96^b^ | 1015.38±44.27^a^ |
|  | 32 | 2,3-Pentanedione | 0^b^ | 5.72±0.14^a^ | 6.3±1.01^a^ | 0^b^ |
|  | 33 | 2-Propanone, 1-hydroxy- | 0^b^ | 20.2±3.23^a^ | 17.25±1.07^a^ | 0^b^ |
|  | 34 | 2-Norcaranone, 3-methyl- | 0^b^ | 4.64±0.12^a^ | 0^b^ | 0^b^ |
|  | 35 | 1(2H)-Naphthalenone, 3,4-dihydro-5,8-dimethyl- | 0^b^ | 0^b^ | 0^b^ | 26.69±5.66^a^ |
| Alcohols | 36 | Ethanol | 1014.3±123.52^c^ | 1415.86±298.04^ab^ | 1073.57±150.19^bc^ | 1732.68±199.31^a^ |
|  | 37 | 1-Propanol, 2-methyl- | 17.61±2.75^c^ | 26.97±1.08^b^ | 24.12±3.64^b^ | 35.83±4.84^a^ |
|  | 38 | 1-Butanol | 10.88±1.25^c^ | 31.8±2.1^a^ | 27.52±4.19^ab^ | 22.86±1.62^b^ |
|  | 39 | 1-Butanol, 3-methyl- | 145.26±17.61^b^ | 174.7±15.62^b^ | 164.1±30.44^b^ | 253.41±27.88^a^ |
|  | 40 | 2-Octanol, (R)- | 1000^a^ | 1000^a^ | 1000^a^ | 1000^a^ |
|  | 41 | 1-Octen-3-ol | 260.05±18.59^a^ | 230.83±21.26^a^ | 236.69±21.21^a^ | 145.79±9.57^b^ |
|  | 42 | 2-Furanmethanol | 108.19±10.68^a^ | 41.97±6.66^b^ | 49.57±3.28^b^ | 0^c^ |
|  | 43 | 1-Propanol, 3-(methylthio)- | 42.69±6.24^b^ | 73.46±10.23^a^ | 62.25±4.28^a^ | 65.4±5.29^a^ |
|  | 44 | Phenylethyl Alcohol | 780.74±78.96^b^ | 963.36±143.63^b^ | 901.16±73.81^b^ | 2085.32±145.48^a^ |
|  | 45 | Estra-1,3,5(10)-trien-17β-ol | 29.35±7.57^a^ | 0^b^ | 23.15±2.17^a^ | 0^b^ |
|  | 46 | 1,2,6-Hexanetriol | 0^b^ | 0^b^ | 9.12±1.14^a^ | 0^b^ |
|  | 47 | β-Ethylphenethyl alcohol | 0^b^ | 0^b^ | 0^b^ | 15.43±2.69^a^ |
| Acids | 48 | Acetic acid | 277.27±20.94^a^ | 235.71±169.77^ab^ | 184.87±71.77^ab^ | 64.57±75.95^b^ |
|  | 49 | Cyclohexan-1,4,5-triol-3-one-1-carboxylic acid | 5.11±0.81^b^ | 59.86±24.12^a^ | 0^b^ | 0^b^ |
|  | 50 | Butanoic acid, 4-hydroxy- | 13.77±1.35^a^ | 8.89±1.9^b^ | 11.53±1.54^a^ | 0^c^ |
|  | 51 | Butanoic acid, 3-methyl- | 41.85±6.17^a^ | 0^b^ | 39.3±1.32^a^ | 0^b^ |
|  | 52 | E-Hex-2-en-1,2-dicarcoxylic acid | 7.18±0.45^a^ | 0^c^ | 6.07±0.23^b^ | 0^c^ |
|  | 53 | Pentanoic acid, 4-methyl- | 13.11±0.38^a^ | 8.88±0.34^c^ | 10.38±0.99^b^ | 0^d^ |
|  | 54 | Hexanoic acid | 19.75±4.16^a^ | 18.93±4.36^a^ | 17.65±3.55^a^ | 0^b^ |
|  | 55 | Nonanoic acid | 22.36±1.1^a^ | 0^b^ | 0^b^ | 0^b^ |
|  | 56 | Pentanoic acid, 3-methyl- | 0^b^ | 43.81±3.85^a^ | 0^b^ | 56.85±13.67^a^ |
|  | 57 | 2-Butenoic acid, 2-methyl- | 0^c^ | 12.96±1.94^a^ | 11.93±1.4^a^ | 8.77±0.94^b^ |
|  | 58 | Propanoic acid, 2-methyl- | 0^b^ | 0^b^ | 0^b^ | 9.73±1.11^a^ |
|  | 59 | Dodecanoic acid, 3-hydroxy- | 0^b^ | 0^b^ | 0^b^ | 7.4±1.79^a^ |
| Pyrazines | 60 | Pyrazine, 2,5-dimethyl- | 13.13±1.51^a^ | 11.92±0.6^a^ | 13.59±1.75^a^ | 0^b^ |
|  | 61 | Pyrazine, 2,6-dimethyl- | 65.7±8.5^a^ | 51.34±4.53^b^ | 58.15±7.75^ab^ | 7±1.52^c^ |
|  | 62 | Pyrazine, ethyl- | 2.7±0.13^b^ | 0^c^ | 2.94±0.08^a^ | 0^c^ |
|  | 63 | Pyrazine, 2-ethyl-6-methyl- | 12.72±1.6^b^ | 17.34±1.62^a^ | 17.5±2.41^a^ | 7.45±0.9^c^ |
|  | 64 | Pyrazine, trimethyl- | 43.59±8.54^a^ | 38.8±2.41^a^ | 40.29±5.07^a^ | 0^b^ |
|  | 65 | Pyrazine, 2-methyl-5-propyl- | 11.24±6.15^a^ | 0^b^ | 6.17±2.15^a^ | 0^b^ |
|  | 66 | Pyrazine, 2-ethyl-3,5-dimethyl- | 4.81±0.47^b^ | 7.4±1.04^a^ | 8.05±0.99^a^ | 8.1±1.72^a^ |
|  | 67 | Pyrazine, 2-ethenyl-6-methyl- | 6.43±0.42^a^ | 5.59±0.54^b^ | 6.66±0.27^a^ | 0^c^ |
|  | 68 | Pyrazine, methyl- | 0^b^ | 0^b^ | 16.49±1.21^a^ | 0^b^ |
| Aldehydes | 69 | Acetaldehyde | 6.35±1.84^b^ | 7.96±0.56^b^ | 8.17±0.87^b^ | 25.71±1.93^a^ |
|  | 70 | Butanal, 2-methyl- | 50.78±4.21^ab^ | 45.36±3.81^ab^ | 54.69±8.52^a^ | 41.11±1.9^b^ |
|  | 71 | Butanal, 3-methyl- | 51.58±4.5^b^ | 53.38±3.29^b^ | 61.16±7.91^ab^ | 69.47±3.37^a^ |
|  | 72 | 2-Butenal, 2-methyl-, (E)- | 4.45±0.26^a^ | 0^c^ | 2.08±0.63^b^ | 3.11±1.51^ab^ |
|  | 73 | Octanal | 4.89±0.34^b^ | 0^c^ | 6.79±0.74^a^ | 0^c^ |
|  | 74 | Nonanal | 16.97±1.59^a^ | 13.66±3.97^a^ | 17.93±1.67^a^ | 12.63±7.34^a^ |
|  | 75 | 3-Furaldehyde | 71.55±8.85^b^ | 35.78±3^d^ | 50.49±5.1^c^ | 95.62±9.57^a^ |
|  | 76 | Decanal | 197.15±7.88^a^ | 120.43±18.67^b^ | 152.48±39.77^ab^ | 152.46±36.07^ab^ |
|  | 77 | Benzaldehyde | 210.93±8.55^b^ | 115.06±5.74^d^ | 146.5±10.17^c^ | 426.7±22.66^a^ |
|  | 78 | Benzeneacetaldehyde | 481.39±118.73^a^ | 518.55±83.37^a^ | 503.57±137.76^a^ | 435.34±38.02^a^ |
|  | 79 | Benzeneacetaldehyde, α-ethylidene- | 27.17±2.03^a^ | 0^b^ | 0^b^ | 0^b^ |
|  | 80 | Benzeneacetaldehyde, α-(2-methylpropylidene)- | 5.41±0.21^ab^ | 4.91±2.6^b^ | 8.04±1.02^a^ | 0^c^ |
|  | 81 | 5-Methyl-2-phenyl-2-hexenal | 8.14±0.31^ab^ | 5.02±0.64^b^ | 11.86±5.13^a^ | 13.44±2.05^a^ |
|  | 82 | 4,4-Dimethylpent-2-enal | 0^c^ | 4.78±0.06^b^ | 4.58±0.66^b^ | 20.39±1.69^a^ |
|  | 83 | 1H-Indene-4-carboxaldehyde, 2,3-dihydro- | 0^c^ | 74.61±12.88^b^ | 97.64±6.1^b^ | 506.14±53.76^a^ |
|  | 84 | 10-Octadecenal | 0^b^ | 0^b^ | 6.54±1.1^a^ | 0^b^ |
|  | 85 | 2-Butenal, (Z)- | 0^b^ | 0^b^ | 0^b^ | 37.45±2.11^a^ |
|  | 86 | 2-Furancarboxaldehyde, 5-methyl- | 0^b^ | 0^b^ | 0^b^ | 9.99±0.38^a^ |
|  | 87 | Propanal, 2-methyl- | 0^b^ | 0^b^ | 0^b^ | 21.93±4.38^a^ |
| Phenols | 88 | Phenol, 2-methoxy- | 17.88±1.31^b^ | 18.51±1.15^ab^ | 20.95±1.77^a^ | 16.31±0.91^b^ |
|  | 89 | Maltol | 132.13±15.07^ab^ | 145.77±29.79^a^ | 168.49±25.71^a^ | 94.11±12.61^b^ |
|  | 90 | Phenol, 4-ethyl-2-methoxy- | 1995.14±158.12^a^ | 1992.24±268.81^a^ | 1961.21±196.1^a^ | 1712.81±213.8^a^ |
|  | 91 | Phenol, 4-ethyl- | 362.94±33.62^a^ | 371.51±52.1^a^ | 362.16±40.1^a^ | 164.44±21.94^b^ |
|  | 92 | 2-Methoxy-4-vinylphenol | 43.11±2.64^a^ | 42.21±9.46^a^ | 46.62±2.49^a^ | 39.74±4.24^a^ |
|  | 93 | 2,4-Di-tert-butylphenol | 464.81±55.44^a^ | 270.59±65.07^b^ | 452.37±89.67^a^ | 414.34±86.62^a^ |
|  | 94 | 2-Naphthalenol | 0^b^ | 20.9±2.62^a^ | 0^b^ | 0^b^ |
| Others | 95 | (2-Aziridinylethyl) amine | 9.9±3.2^ab^ | 9.11±2.33^b^ | 6.71±2.89^b^ | 14.99±3.35^a^ |
|  | 96 | Pentanoic acid, 2-methyl-, anhydride | 89.26±5.02^a^ | 82.31±21.39^a^ | 69.58±17.76^a^ | 0^b^ |
|  | 97 | Furan, tetrahydro-2,2-dimethyl-5-(1-methylpropyl)- | 55.28±6.86^a^ | 0^b^ | 0^b^ | 0^b^ |
|  | 98 | 3-Acetyl-1H-pyrroline | 66.55±8.31^a^ | 0^b^ | 73.42±0.08^a^ | 0^b^ |
|  | 99 | Ethanone, 1-(1H-pyrrol-2-yl)- | 0^c^ | 53.53±4.35^a^ | 0^c^ | 26.24±0.78^b^ |
|  | 100 | α-[5-Ethyl-2,3,4,5-tetrahydro-2-furyl] glycine | 0^b^ | 0^b^ | 42.61±4.34^a^ | 0^b^ |

**Table S2** Statistical analysis of soy sauce sample data sequencing

| Sample | Valid sequence | | High-quality sequence | | Proportion（%） | |
| --- | --- | --- | --- | --- | --- | --- |
|  | Bacteria | Fungi | Bacteria | Fungi | Bacteria | Fungi |
| SMC-L1 | 86198 | 105407 | 78840 | 94753 | 91.46% | 89.89% |
| SMC-L2 | 76381 | 126024 | 72309 | 99663 | 94.67% | 80.08% |
| SMC-L3 | 87483 | 90760 | 72397 | 84131 | 82.76% | 92.70% |
| HS | 83121 | 106809 | 75936 | 92485 | 91.36% | 86.59% |

**Table S3** Richness and diversity of bacteria and fungi in different samples

| Sample | Chao1 | | Observed species | | Simpson | | Shannon | |
| --- | --- | --- | --- | --- | --- | --- | --- | --- |
|  | Bacteria | Fungi | Bacteria | Fungi | Bacteria | Fungi | Bacteria | Fungi |
| SMC-L1 | 114 | 34 | 113 | 34 | 0.77 | 0.55 | 2.81 | 1.94 |
| SMC-L2 | 133 | 51 | 133 | 51 | 0.75 | 0.72 | 3.09 | 2.51 |
| SMC-L3 | 222.1 | 44 | 222 | 44 | 0.77 | 0.40 | 3.37 | 1.62 |
| HS | 99.5 | 51.17 | 99 | 51 | 0.84 | 0.74 | 3.59 | 2.35 |

**Table S4** Complete Pairwise Correlation Results between Microbes and Metabolites, Including Spearman's ρ and FDR-adjusted q-values

| q-value | N1 | N2 | N3 | N4 | N5 | N6 | N7 | N8 | N9 | N10 | N11 | N12 | N13 | N14 | N15 | N16 | N17 | N18 | N19 | N20 | N21 | N22 | N23 | N24 | N25 | N26 | N27 | N28 | N29 | N30 | N31 | N32 | N33 | N34 | N35 | N36 | N37 | N38 | N39 | N40 | N41 | N42 | N43 | N44 | N45 | N46 | N47 | N48 | N49 | N50 | N51 | N52 | N53 | N54 | N55 |
| --- | --- | --- | --- | --- | --- | --- | --- | --- | --- | --- | --- | --- | --- | --- | --- | --- | --- | --- | --- | --- | --- | --- | --- | --- | --- | --- | --- | --- | --- | --- | --- | --- | --- | --- | --- | --- | --- | --- | --- | --- | --- | --- | --- | --- | --- | --- | --- | --- | --- | --- | --- | --- | --- | --- | --- |
| *Weissella* | 0.80 | 0.60 | 0.68 | 0.60 | 0.40 | 0.60 | 1.00 | 0.60 | 0.80 | 0.20 | 0.00 | 0.20 | 0.60 | 1.00 | 0.60 | 0.60 | 0.60 | 0.60 | 0.60 | 0.60 | 0.20 | 0.60 | 0.80 | 0.80 | 0.40 | 0.60 | 0.80 | 0.60 | 0.60 | 0.20 | 0.40 | 0.20 | 0.60 | 1.00 | 0.80 | 0.20 | 0.20 | 0.20 | 0.20 | 0.80 | 0.60 | 0.60 | 0.60 | 0.20 | 0.60 | 0.40 | 0.60 | 0.37 | 0.79 | 0.40 | 0.40 | 0.80 | 0.60 | 1.00 | 0.20 |
| *Staphylococcus* | 0.20 | 0.00 | 0.05 | 0.00 | 0.60 | 0.00 | 0.20 | 0.00 | 0.20 | 0.80 | 0.60 | 0.20 | 0.00 | 0.20 | 0.00 | 0.60 | 0.60 | 0.60 | 0.60 | 0.60 | 0.80 | 0.60 | 0.20 | 0.20 | 0.60 | 0.40 | 0.20 | 0.60 | 0.60 | 0.80 | 0.60 | 0.80 | 0.00 | 0.20 | 0.20 | 0.80 | 0.20 | 0.80 | 0.80 | 0.20 | 0.00 | 0.00 | 0.00 | 0.20 | 0.00 | 0.60 | 0.00 | 0.05 | 0.05 | 0.60 | 0.60 | 0.20 | 0.60 | 0.20 | 0.20 |
| *Tetragenococcus* | 0.80 | 0.60 | 0.68 | 0.60 | 0.40 | 0.60 | 1.00 | 0.60 | 0.80 | 0.20 | 0.00 | 0.20 | 0.60 | 1.00 | 0.60 | 0.60 | 0.60 | 0.60 | 0.60 | 0.60 | 0.20 | 0.60 | 0.80 | 0.80 | 0.40 | 0.60 | 0.80 | 0.60 | 0.60 | 0.20 | 0.40 | 0.20 | 0.60 | 1.00 | 0.80 | 0.20 | 0.20 | 0.20 | 0.20 | 0.80 | 0.60 | 0.60 | 0.60 | 0.20 | 0.60 | 0.40 | 0.60 | 0.37 | 0.79 | 0.40 | 0.40 | 0.80 | 0.60 | 1.00 | 0.20 |
| *Leuconostoc* | 0.20 | 0.00 | 0.05 | 0.00 | 0.60 | 0.00 | 0.20 | 0.00 | 0.20 | 0.80 | 0.60 | 0.20 | 0.00 | 0.20 | 0.00 | 0.60 | 0.60 | 0.60 | 0.60 | 0.60 | 0.80 | 0.60 | 0.20 | 0.20 | 0.60 | 0.40 | 0.20 | 0.60 | 0.60 | 0.80 | 0.60 | 0.80 | 0.00 | 0.20 | 0.20 | 0.80 | 0.20 | 0.80 | 0.80 | 0.20 | 0.00 | 0.00 | 0.00 | 0.20 | 0.00 | 0.60 | 0.00 | 0.05 | 0.05 | 0.60 | 0.60 | 0.20 | 0.60 | 0.20 | 0.20 |
| *Corynebacterium* | 0.00 | 0.20 | 0.05 | 0.20 | 0.80 | 0.20 | 0.60 | 0.20 | 0.00 | 0.60 | 0.80 | 0.60 | 0.20 | 0.60 | 0.20 | 0.20 | 0.20 | 0.20 | 0.20 | 0.20 | 0.60 | 0.20 | 0.00 | 0.00 | 0.80 | 1.00 | 0.00 | 0.20 | 0.20 | 0.60 | 0.80 | 0.60 | 0.20 | 0.60 | 0.00 | 0.60 | 0.60 | 0.60 | 0.60 | 0.00 | 0.20 | 0.20 | 0.20 | 0.60 | 0.20 | 0.80 | 0.20 | 0.37 | 0.37 | 0.80 | 0.80 | 0.00 | 0.20 | 0.60 | 0.60 |
| *Pseudomonas* | 0.80 | 0.60 | 0.68 | 0.60 | 0.00 | 0.60 | 0.20 | 0.60 | 0.80 | 0.20 | 0.40 | 1.00 | 0.60 | 0.20 | 0.60 | 0.60 | 0.60 | 0.60 | 0.60 | 0.60 | 0.20 | 0.60 | 0.80 | 0.80 | 0.00 | 0.60 | 0.80 | 0.60 | 0.60 | 0.20 | 0.00 | 0.20 | 0.60 | 0.20 | 0.80 | 0.20 | 1.00 | 0.20 | 0.20 | 0.80 | 0.60 | 0.60 | 0.60 | 1.00 | 0.60 | 0.00 | 0.60 | 0.79 | 0.37 | 0.00 | 0.00 | 0.80 | 0.60 | 0.20 | 1.00 |
| *Enterococcus* | 0.20 | 0.60 | 0.37 | 0.60 | 0.60 | 0.60 | 0.80 | 0.60 | 0.20 | 1.00 | 0.60 | 0.80 | 0.60 | 0.80 | 0.60 | 0.00 | 0.00 | 0.00 | 0.00 | 0.00 | 0.20 | 0.00 | 0.20 | 0.20 | 0.60 | 0.60 | 0.20 | 0.00 | 0.00 | 1.00 | 0.60 | 0.20 | 0.60 | 0.80 | 0.20 | 0.20 | 0.80 | 0.20 | 0.20 | 0.20 | 0.60 | 0.60 | 0.60 | 0.80 | 0.60 | 0.60 | 0.60 | 0.89 | 0.68 | 0.60 | 0.60 | 0.20 | 0.00 | 0.80 | 0.80 |
| *Bacillus* | 0.68 | 0.37 | 0.50 | 0.37 | 0.68 | 0.37 | 0.68 | 0.37 | 0.68 | 0.37 | 0.05 | 0.05 | 0.37 | 0.68 | 0.37 | 0.68 | 0.68 | 0.68 | 0.68 | 0.68 | 0.37 | 0.68 | 0.68 | 0.68 | 0.68 | 0.37 | 0.68 | 0.68 | 0.68 | 0.37 | 0.68 | 0.37 | 0.37 | 0.68 | 0.68 | 0.37 | 0.05 | 0.37 | 0.37 | 0.68 | 0.37 | 0.37 | 0.37 | 0.05 | 0.37 | 0.68 | 0.37 | 0.17 | 0.50 | 0.68 | 0.68 | 0.68 | 0.68 | 0.68 | 0.05 |
| *Lactococcus* | 0.20 | 0.00 | 0.05 | 0.00 | 0.60 | 0.00 | 0.20 | 0.00 | 0.20 | 0.80 | 0.60 | 0.20 | 0.00 | 0.20 | 0.00 | 0.60 | 0.60 | 0.60 | 0.60 | 0.60 | 0.80 | 0.60 | 0.20 | 0.20 | 0.60 | 0.40 | 0.20 | 0.60 | 0.60 | 0.80 | 0.60 | 0.80 | 0.00 | 0.20 | 0.20 | 0.80 | 0.20 | 0.80 | 0.80 | 0.20 | 0.00 | 0.00 | 0.00 | 0.20 | 0.00 | 0.60 | 0.00 | 0.05 | 0.05 | 0.60 | 0.60 | 0.20 | 0.60 | 0.20 | 0.20 |
| *Kurthia* | 0.05 | 0.05 | 0.00 | 0.05 | 0.68 | 0.05 | 0.37 | 0.05 | 0.05 | 0.68 | 0.68 | 0.37 | 0.05 | 0.37 | 0.05 | 0.37 | 0.37 | 0.37 | 0.37 | 0.37 | 0.68 | 0.37 | 0.05 | 0.05 | 0.68 | 0.68 | 0.05 | 0.37 | 0.37 | 0.68 | 0.68 | 0.68 | 0.05 | 0.37 | 0.05 | 0.68 | 0.37 | 0.68 | 0.68 | 0.05 | 0.05 | 0.05 | 0.05 | 0.37 | 0.05 | 0.68 | 0.05 | 0.17 | 0.17 | 0.68 | 0.68 | 0.05 | 0.37 | 0.37 | 0.37 |
| *Aspergillus* | 0.60 | 0.20 | 0.37 | 0.20 | 0.20 | 0.20 | 0.00 | 0.20 | 0.60 | 0.60 | 1.00 | 0.40 | 0.20 | 0.00 | 0.20 | 0.80 | 0.80 | 0.80 | 0.80 | 0.80 | 0.60 | 0.80 | 0.60 | 0.60 | 0.20 | 0.20 | 0.60 | 0.80 | 0.80 | 0.60 | 0.20 | 0.60 | 0.20 | 0.00 | 0.60 | 0.60 | 0.40 | 0.60 | 0.60 | 0.60 | 0.20 | 0.20 | 0.20 | 0.40 | 0.20 | 0.20 | 0.20 | 0.26 | 0.05 | 0.20 | 0.20 | 0.60 | 0.80 | 0.00 | 0.40 |
| *Wickerhamiella* | 0.20 | 0.60 | 0.37 | 0.60 | 0.60 | 0.60 | 0.80 | 0.60 | 0.20 | 1.00 | 0.60 | 0.80 | 0.60 | 0.80 | 0.60 | 0.00 | 0.00 | 0.00 | 0.00 | 0.00 | 0.20 | 0.00 | 0.20 | 0.20 | 0.60 | 0.60 | 0.20 | 0.00 | 0.00 | 1.00 | 0.60 | 0.20 | 0.60 | 0.80 | 0.20 | 0.20 | 0.80 | 0.20 | 0.20 | 0.20 | 0.60 | 0.60 | 0.60 | 0.80 | 0.60 | 0.60 | 0.60 | 0.89 | 0.68 | 0.60 | 0.60 | 0.20 | 0.00 | 0.80 | 0.80 |
| *Thermoascus* | 0.37 | 0.68 | 0.50 | 0.68 | 0.37 | 0.68 | 0.68 | 0.68 | 0.37 | 0.68 | 0.37 | 0.68 | 0.68 | 0.68 | 0.68 | 0.05 | 0.05 | 0.05 | 0.05 | 0.05 | 0.05 | 0.05 | 0.37 | 0.37 | 0.37 | 0.68 | 0.37 | 0.05 | 0.05 | 0.68 | 0.37 | 0.05 | 0.68 | 0.68 | 0.37 | 0.05 | 0.68 | 0.05 | 0.05 | 0.37 | 0.68 | 0.68 | 0.68 | 0.68 | 0.68 | 0.37 | 0.68 | 1.00 | 0.67 | 0.37 | 0.37 | 0.37 | 0.05 | 0.68 | 0.68 |
| *Rhodotorula* | 0.00 | 0.20 | 0.05 | 0.20 | 0.80 | 0.20 | 0.60 | 0.20 | 0.00 | 0.60 | 0.80 | 0.60 | 0.20 | 0.60 | 0.20 | 0.20 | 0.20 | 0.20 | 0.20 | 0.20 | 0.60 | 0.20 | 0.00 | 0.00 | 0.80 | 1.00 | 0.00 | 0.20 | 0.20 | 0.60 | 0.80 | 0.60 | 0.20 | 0.60 | 0.00 | 0.60 | 0.60 | 0.60 | 0.60 | 0.00 | 0.20 | 0.20 | 0.20 | 0.60 | 0.20 | 0.80 | 0.20 | 0.37 | 0.37 | 0.80 | 0.80 | 0.00 | 0.20 | 0.60 | 0.60 |
| *Starmerella* | 0.05 | 0.37 | 0.17 | 0.37 | 0.68 | 0.37 | 0.68 | 0.37 | 0.05 | 0.79 | 0.89 | 0.89 | 0.37 | 0.68 | 0.37 | 0.05 | 0.05 | 0.05 | 0.05 | 0.05 | 0.37 | 0.05 | 0.05 | 0.05 | 0.68 | 0.79 | 0.05 | 0.05 | 0.05 | 0.79 | 0.68 | 0.37 | 0.37 | 0.68 | 0.05 | 0.37 | 0.89 | 0.37 | 0.37 | 0.05 | 0.37 | 0.37 | 0.37 | 0.89 | 0.37 | 0.68 | 0.37 | 0.61 | 0.50 | 0.68 | 0.68 | 0.05 | 0.05 | 0.68 | 0.89 |
| *Zygosaccharomyces* | 0.00 | 0.20 | 0.05 | 0.20 | 0.80 | 0.20 | 0.60 | 0.20 | 0.00 | 0.60 | 0.80 | 0.60 | 0.20 | 0.60 | 0.20 | 0.20 | 0.20 | 0.20 | 0.20 | 0.20 | 0.60 | 0.20 | 0.00 | 0.00 | 0.80 | 1.00 | 0.00 | 0.20 | 0.20 | 0.60 | 0.80 | 0.60 | 0.20 | 0.60 | 0.00 | 0.60 | 0.60 | 0.60 | 0.60 | 0.00 | 0.20 | 0.20 | 0.20 | 0.60 | 0.20 | 0.80 | 0.20 | 0.37 | 0.37 | 0.80 | 0.80 | 0.00 | 0.20 | 0.60 | 0.60 |
| *Thermomyces* | 0.74 | 0.74 | 1.00 | 0.74 | 0.74 | 0.74 | 0.74 | 0.74 | 0.74 | 0.74 | 0.23 | 0.23 | 0.74 | 0.74 | 0.74 | 0.23 | 0.23 | 0.23 | 0.23 | 0.23 | 0.23 | 0.23 | 0.74 | 0.74 | 0.74 | 0.23 | 0.74 | 0.23 | 0.23 | 0.74 | 0.74 | 0.23 | 0.74 | 0.74 | 0.74 | 0.23 | 0.23 | 0.23 | 0.23 | 0.74 | 0.74 | 0.74 | 0.74 | 0.23 | 0.74 | 0.74 | 0.74 | 0.46 | 0.73 | 0.74 | 0.74 | 0.74 | 0.23 | 0.74 | 0.23 |
| *Rasamsonia* | 0.74 | 0.74 | 1.00 | 0.74 | 0.74 | 0.74 | 0.74 | 0.74 | 0.74 | 0.74 | 0.23 | 0.23 | 0.74 | 0.74 | 0.74 | 0.23 | 0.23 | 0.23 | 0.23 | 0.23 | 0.23 | 0.23 | 0.74 | 0.74 | 0.74 | 0.23 | 0.74 | 0.23 | 0.23 | 0.74 | 0.74 | 0.23 | 0.74 | 0.74 | 0.74 | 0.23 | 0.23 | 0.23 | 0.23 | 0.74 | 0.74 | 0.74 | 0.74 | 0.23 | 0.74 | 0.74 | 0.74 | 0.46 | 0.73 | 0.74 | 0.74 | 0.74 | 0.23 | 0.74 | 0.23 |
| *Monascus* | 0.05 | 0.37 | 0.17 | 0.37 | 0.89 | 0.37 | 0.89 | 0.37 | 0.05 | 0.37 | 0.68 | 0.68 | 0.37 | 0.89 | 0.37 | 0.26 | 0.26 | 0.26 | 0.26 | 0.26 | 0.79 | 0.26 | 0.05 | 0.05 | 0.89 | 0.79 | 0.05 | 0.26 | 0.26 | 0.37 | 0.89 | 0.79 | 0.37 | 0.89 | 0.05 | 0.79 | 0.68 | 0.79 | 0.79 | 0.05 | 0.37 | 0.37 | 0.37 | 0.68 | 0.37 | 0.89 | 0.37 | 0.50 | 0.61 | 0.89 | 0.89 | 0.05 | 0.26 | 0.89 | 0.68 |
| *Cladosporium* | 0.23 | 0.23 | 0.18 | 0.23 | 0.74 | 0.23 | 0.74 | 0.23 | 0.23 | 0.23 | 0.23 | 0.23 | 0.23 | 0.74 | 0.23 | 0.74 | 0.74 | 0.74 | 0.74 | 0.74 | 0.74 | 0.74 | 0.23 | 0.23 | 0.74 | 0.74 | 0.23 | 0.74 | 0.74 | 0.23 | 0.74 | 0.74 | 0.23 | 0.74 | 0.23 | 0.74 | 0.23 | 0.74 | 0.74 | 0.23 | 0.23 | 0.23 | 0.23 | 0.23 | 0.23 | 0.74 | 0.23 | 0.18 | 0.46 | 0.74 | 0.74 | 0.23 | 0.74 | 0.74 | 0.23 |

| ρ | N1 | N2 | N3 | N4 | N5 | N6 | N7 | N8 | N9 | N10 | N11 | N12 | N13 | N14 | N15 | N16 | N17 | N18 | N19 | N20 | N21 | N22 | N23 | N24 | N25 | N26 | N27 | N28 | N29 | N30 | N31 | N32 | N33 | N34 | N35 | N36 | N37 | N38 | N39 | N40 | N41 | N42 | N43 | N44 | N45 | N46 | N47 | N48 | N49 | N50 | N51 | N52 | N53 | N54 | N55 |
| --- | --- | --- | --- | --- | --- | --- | --- | --- | --- | --- | --- | --- | --- | --- | --- | --- | --- | --- | --- | --- | --- | --- | --- | --- | --- | --- | --- | --- | --- | --- | --- | --- | --- | --- | --- | --- | --- | --- | --- | --- | --- | --- | --- | --- | --- | --- | --- | --- | --- | --- | --- | --- | --- | --- | --- |
| *Weissella* | 0.20 | -0.40 | -0.32 | -0.40 | 0.60 | -0.40 | 0.00 | -0.40 | 0.20 | 0.80 | -1.00 | -0.80 | 0.40 | 0.00 | -0.40 | 0.40 | 0.40 | 0.40 | 0.40 | 0.40 | 0.80 | 0.40 | -0.20 | -0.20 | -0.60 | -0.40 | -0.20 | 0.40 | 0.40 | 0.80 | 0.60 | -0.80 | -0.40 | 0.00 | 0.20 | -0.80 | -0.80 | -0.80 | 0.80 | 0.20 | 0.40 | -0.40 | -0.40 | 0.80 | 0.40 | 0.60 | 0.40 | -0.63 | 0.21 | 0.60 | 0.60 | -0.20 | 0.40 | 0.00 | -0.80 |
| *Staphylococcus* | 0.80 | -1.00 | -0.95 | -1.00 | -0.40 | -1.00 | -0.80 | -1.00 | 0.80 | 0.20 | -0.40 | -0.80 | 1.00 | -0.80 | -1.00 | -0.40 | -0.40 | -0.40 | -0.40 | -0.40 | -0.20 | -0.40 | -0.80 | -0.80 | 0.40 | -0.60 | -0.80 | -0.40 | -0.40 | 0.20 | -0.40 | 0.20 | -1.00 | -0.80 | 0.80 | 0.20 | -0.80 | 0.20 | -0.20 | 0.80 | 1.00 | -1.00 | -1.00 | 0.80 | 1.00 | -0.40 | 1.00 | -0.95 | 0.95 | -0.40 | -0.40 | -0.80 | -0.40 | -0.80 | -0.80 |
| *Tetragenococcus* | -0.20 | 0.40 | 0.32 | 0.40 | -0.60 | 0.40 | 0.00 | 0.40 | -0.20 | -0.80 | 1.00 | 0.80 | -0.40 | 0.00 | 0.40 | -0.40 | -0.40 | -0.40 | -0.40 | -0.40 | -0.80 | -0.40 | 0.20 | 0.20 | 0.60 | 0.40 | 0.20 | -0.40 | -0.40 | -0.80 | -0.60 | 0.80 | 0.40 | 0.00 | -0.20 | 0.80 | 0.80 | 0.80 | -0.80 | -0.20 | -0.40 | 0.40 | 0.40 | -0.80 | -0.40 | -0.60 | -0.40 | 0.63 | -0.21 | -0.60 | -0.60 | 0.20 | -0.40 | 0.00 | 0.80 |
| *Leuconostoc* | 0.80 | -1.00 | -0.95 | -1.00 | -0.40 | -1.00 | -0.80 | -1.00 | 0.80 | 0.20 | -0.40 | -0.80 | 1.00 | -0.80 | -1.00 | -0.40 | -0.40 | -0.40 | -0.40 | -0.40 | -0.20 | -0.40 | -0.80 | -0.80 | 0.40 | -0.60 | -0.80 | -0.40 | -0.40 | 0.20 | -0.40 | 0.20 | -1.00 | -0.80 | 0.80 | 0.20 | -0.80 | 0.20 | -0.20 | 0.80 | 1.00 | -1.00 | -1.00 | 0.80 | 1.00 | -0.40 | 1.00 | -0.95 | 0.95 | -0.40 | -0.40 | -0.80 | -0.40 | -0.80 | -0.80 |
| *Corynebacterium* | 1.00 | -0.80 | -0.95 | -0.80 | -0.20 | -0.80 | -0.40 | -0.80 | 1.00 | 0.40 | -0.20 | -0.40 | 0.80 | -0.40 | -0.80 | -0.80 | -0.80 | -0.80 | -0.80 | -0.80 | -0.40 | -0.80 | -1.00 | -1.00 | 0.20 | 0.00 | -1.00 | -0.80 | -0.80 | 0.40 | -0.20 | 0.40 | -0.80 | -0.40 | 1.00 | 0.40 | -0.40 | 0.40 | -0.40 | 1.00 | 0.80 | -0.80 | -0.80 | 0.40 | 0.80 | -0.20 | 0.80 | -0.63 | 0.63 | -0.20 | -0.20 | -1.00 | -0.80 | -0.40 | -0.40 |
| *Pseudomonas* | -0.20 | 0.40 | 0.32 | 0.40 | 1.00 | 0.40 | 0.80 | 0.40 | -0.20 | 0.80 | -0.60 | 0.00 | -0.40 | 0.80 | 0.40 | 0.40 | 0.40 | 0.40 | 0.40 | 0.40 | 0.80 | 0.40 | 0.20 | 0.20 | -1.00 | 0.40 | 0.20 | 0.40 | 0.40 | 0.80 | 1.00 | -0.80 | 0.40 | 0.80 | -0.20 | -0.80 | 0.00 | -0.80 | 0.80 | -0.20 | -0.40 | 0.40 | 0.40 | 0.00 | -0.40 | 1.00 | -0.40 | 0.21 | -0.63 | 1.00 | 1.00 | 0.20 | 0.40 | 0.80 | 0.00 |
| *Enterococcus* | 0.80 | -0.40 | -0.63 | -0.40 | -0.40 | -0.40 | -0.20 | -0.40 | 0.80 | 0.00 | 0.40 | 0.20 | 0.40 | -0.20 | -0.40 | -1.00 | -1.00 | -1.00 | -1.00 | -1.00 | -0.80 | -1.00 | -0.80 | -0.80 | 0.40 | 0.40 | -0.80 | -1.00 | -1.00 | 0.00 | -0.40 | 0.80 | -0.40 | -0.20 | 0.80 | 0.80 | 0.20 | 0.80 | -0.80 | 0.80 | 0.40 | -0.40 | -0.40 | -0.20 | 0.40 | -0.40 | 0.40 | -0.11 | 0.32 | -0.40 | -0.40 | -0.80 | -1.00 | -0.20 | 0.20 |
| *Bacillus* | 0.32 | -0.63 | -0.50 | -0.63 | 0.32 | -0.63 | -0.32 | -0.63 | 0.32 | 0.63 | -0.95 | -0.95 | 0.63 | -0.32 | -0.63 | 0.32 | 0.32 | 0.32 | 0.32 | 0.32 | 0.63 | 0.32 | -0.32 | -0.32 | -0.32 | -0.63 | -0.32 | 0.32 | 0.32 | 0.63 | 0.32 | -0.63 | -0.63 | -0.32 | 0.32 | -0.63 | -0.95 | -0.63 | 0.63 | 0.32 | 0.63 | -0.63 | -0.63 | 0.95 | 0.63 | 0.32 | 0.63 | -0.83 | 0.50 | 0.32 | 0.32 | -0.32 | 0.32 | -0.32 | -0.95 |
| *Lactococcus* | 0.80 | -1.00 | -0.95 | -1.00 | -0.40 | -1.00 | -0.80 | -1.00 | 0.80 | 0.20 | -0.40 | -0.80 | 1.00 | -0.80 | -1.00 | -0.40 | -0.40 | -0.40 | -0.40 | -0.40 | -0.20 | -0.40 | -0.80 | -0.80 | 0.40 | -0.60 | -0.80 | -0.40 | -0.40 | 0.20 | -0.40 | 0.20 | -1.00 | -0.80 | 0.80 | 0.20 | -0.80 | 0.20 | -0.20 | 0.80 | 1.00 | -1.00 | -1.00 | 0.80 | 1.00 | -0.40 | 1.00 | -0.95 | 0.95 | -0.40 | -0.40 | -0.80 | -0.40 | -0.80 | -0.80 |
| *Kurthia* | 0.95 | -0.95 | -1.00 | -0.95 | -0.32 | -0.95 | -0.63 | -0.95 | 0.95 | 0.32 | -0.32 | -0.63 | 0.95 | -0.63 | -0.95 | -0.63 | -0.63 | -0.63 | -0.63 | -0.63 | -0.32 | -0.63 | -0.95 | -0.95 | 0.32 | -0.32 | -0.95 | -0.63 | -0.63 | 0.32 | -0.32 | 0.32 | -0.95 | -0.63 | 0.95 | 0.32 | -0.63 | 0.32 | -0.32 | 0.95 | 0.95 | -0.95 | -0.95 | 0.63 | 0.95 | -0.32 | 0.95 | -0.83 | 0.83 | -0.32 | -0.32 | -0.95 | -0.63 | -0.63 | -0.63 |
| *Aspergillus* | -0.40 | 0.80 | 0.63 | 0.80 | 0.80 | 0.80 | 1.00 | 0.80 | -0.40 | 0.40 | 0.00 | 0.60 | -0.80 | 1.00 | 0.80 | 0.20 | 0.20 | 0.20 | 0.20 | 0.20 | 0.40 | 0.20 | 0.40 | 0.40 | -0.80 | 0.80 | 0.40 | 0.20 | 0.20 | 0.40 | 0.80 | -0.40 | 0.80 | 1.00 | -0.40 | -0.40 | 0.60 | -0.40 | 0.40 | -0.40 | -0.80 | 0.80 | 0.80 | -0.60 | -0.80 | 0.80 | -0.80 | 0.74 | -0.95 | 0.80 | 0.80 | 0.40 | 0.20 | 1.00 | 0.60 |
| *Wickerhamiella* | 0.80 | -0.40 | -0.63 | -0.40 | -0.40 | -0.40 | -0.20 | -0.40 | 0.80 | 0.00 | 0.40 | 0.20 | 0.40 | -0.20 | -0.40 | -1.00 | -1.00 | -1.00 | -1.00 | -1.00 | -0.80 | -1.00 | -0.80 | -0.80 | 0.40 | 0.40 | -0.80 | -1.00 | -1.00 | 0.00 | -0.40 | 0.80 | -0.40 | -0.20 | 0.80 | 0.80 | 0.20 | 0.80 | -0.80 | 0.80 | 0.40 | -0.40 | -0.40 | -0.20 | 0.40 | -0.40 | 0.40 | -0.11 | 0.32 | -0.40 | -0.40 | -0.80 | -1.00 | -0.20 | 0.20 |
| *Thermoascus* | -0.63 | 0.32 | 0.50 | 0.32 | 0.63 | 0.32 | 0.32 | 0.32 | -0.63 | 0.32 | -0.63 | -0.32 | -0.32 | 0.32 | 0.32 | 0.95 | 0.95 | 0.95 | 0.95 | 0.95 | 0.95 | 0.95 | 0.63 | 0.63 | -0.63 | -0.32 | 0.63 | 0.95 | 0.95 | 0.32 | 0.63 | -0.95 | 0.32 | 0.32 | -0.63 | -0.95 | -0.32 | -0.95 | 0.95 | -0.63 | -0.32 | 0.32 | 0.32 | 0.32 | -0.32 | 0.63 | -0.32 | 0.00 | -0.33 | 0.63 | 0.63 | 0.63 | 0.95 | 0.32 | -0.32 |
| *Rhodotorula* | -1.00 | 0.80 | 0.95 | 0.80 | 0.20 | 0.80 | 0.40 | 0.80 | -1.00 | -0.40 | 0.20 | 0.40 | -0.80 | 0.40 | 0.80 | 0.80 | 0.80 | 0.80 | 0.80 | 0.80 | 0.40 | 0.80 | 1.00 | 1.00 | -0.20 | 0.00 | 1.00 | 0.80 | 0.80 | -0.40 | 0.20 | -0.40 | 0.80 | 0.40 | -1.00 | -0.40 | 0.40 | -0.40 | 0.40 | -1.00 | -0.80 | 0.80 | 0.80 | -0.40 | -0.80 | 0.20 | -0.80 | 0.63 | -0.63 | 0.20 | 0.20 | 1.00 | 0.80 | 0.40 | 0.40 |
| *Starmerella* | 0.95 | -0.63 | -0.83 | -0.63 | -0.32 | -0.63 | -0.32 | -0.63 | 0.95 | 0.21 | 0.11 | -0.11 | 0.63 | -0.32 | -0.63 | -0.95 | -0.95 | -0.95 | -0.95 | -0.95 | -0.63 | -0.95 | -0.95 | -0.95 | 0.32 | 0.21 | -0.95 | -0.95 | -0.95 | 0.21 | -0.32 | 0.63 | -0.63 | -0.32 | 0.95 | 0.63 | -0.11 | 0.63 | -0.63 | 0.95 | 0.63 | -0.63 | -0.63 | 0.11 | 0.63 | -0.32 | 0.63 | -0.39 | 0.50 | -0.32 | -0.32 | -0.95 | -0.95 | -0.32 | -0.11 |
| *Zygosaccharomyces* | 1.00 | -0.80 | -0.95 | -0.80 | -0.20 | -0.80 | -0.40 | -0.80 | 1.00 | 0.40 | -0.20 | -0.40 | 0.80 | -0.40 | -0.80 | -0.80 | -0.80 | -0.80 | -0.80 | -0.80 | -0.40 | -0.80 | -1.00 | -1.00 | 0.20 | 0.00 | -1.00 | -0.80 | -0.80 | 0.40 | -0.20 | 0.40 | -0.80 | -0.40 | 1.00 | 0.40 | -0.40 | 0.40 | -0.40 | 1.00 | 0.80 | -0.80 | -0.80 | 0.40 | 0.80 | -0.20 | 0.80 | -0.63 | 0.63 | -0.20 | -0.20 | -1.00 | -0.80 | -0.40 | -0.40 |
| *Thermomyces* | -0.26 | -0.26 | 0.00 | -0.26 | 0.26 | -0.26 | -0.26 | -0.26 | -0.26 | 0.26 | -0.77 | -0.77 | 0.26 | -0.26 | -0.26 | 0.77 | 0.77 | 0.77 | 0.77 | 0.77 | 0.77 | 0.77 | 0.26 | 0.26 | -0.26 | -0.77 | 0.26 | 0.77 | 0.77 | 0.26 | 0.26 | -0.77 | -0.26 | -0.26 | -0.26 | -0.77 | -0.77 | -0.77 | 0.77 | -0.26 | 0.26 | -0.26 | -0.26 | 0.77 | 0.26 | 0.26 | 0.26 | -0.54 | 0.27 | 0.26 | 0.26 | 0.26 | 0.77 | -0.26 | -0.77 |
| *Rasamsonia* | -0.26 | -0.26 | 0.00 | -0.26 | 0.26 | -0.26 | -0.26 | -0.26 | -0.26 | 0.26 | -0.77 | -0.77 | 0.26 | -0.26 | -0.26 | 0.77 | 0.77 | 0.77 | 0.77 | 0.77 | 0.77 | 0.77 | 0.26 | 0.26 | -0.26 | -0.77 | 0.26 | 0.77 | 0.77 | 0.26 | 0.26 | -0.77 | -0.26 | -0.26 | -0.26 | -0.77 | -0.77 | -0.77 | 0.77 | -0.26 | 0.26 | -0.26 | -0.26 | 0.77 | 0.26 | 0.26 | 0.26 | -0.54 | 0.27 | 0.26 | 0.26 | 0.26 | 0.77 | -0.26 | -0.77 |
| *Monascus* | -0.95 | 0.63 | 0.83 | 0.63 | -0.11 | 0.63 | 0.11 | 0.63 | -0.95 | -0.63 | 0.32 | 0.32 | -0.63 | 0.11 | 0.63 | 0.74 | 0.74 | 0.74 | 0.74 | 0.74 | 0.21 | 0.74 | 0.95 | 0.95 | 0.11 | -0.21 | 0.95 | 0.74 | 0.74 | -0.63 | -0.11 | -0.21 | 0.63 | 0.11 | -0.95 | -0.21 | 0.32 | -0.21 | 0.21 | -0.95 | -0.63 | 0.63 | 0.63 | -0.32 | -0.63 | -0.11 | -0.63 | 0.50 | -0.39 | -0.11 | -0.11 | 0.95 | 0.74 | 0.11 | 0.32 |
| *Cladosporium* | -0.77 | 0.77 | 0.82 | 0.77 | -0.26 | 0.77 | 0.26 | 0.77 | -0.77 | -0.77 | 0.77 | 0.77 | -0.77 | 0.26 | 0.77 | 0.26 | 0.26 | 0.26 | 0.26 | 0.26 | -0.26 | 0.26 | 0.77 | 0.77 | 0.26 | 0.26 | 0.77 | 0.26 | 0.26 | -0.77 | -0.26 | 0.26 | 0.77 | 0.26 | -0.77 | 0.26 | 0.77 | 0.26 | -0.26 | -0.77 | -0.77 | 0.77 | 0.77 | -0.77 | -0.77 | -0.26 | -0.77 | 0.82 | -0.54 | -0.26 | -0.26 | 0.77 | 0.26 | 0.26 | 0.77 |

**Table S4 (continue)** Correspondence between Metabolites and Their Codes

| Numbers | Metabolites |
| --- | --- |
| N1 | Amino acid nitrogen |
| N2 | Total acidity |
| N3 | Reducing sugar |
| N4 | Total nitrogen |
| N5 | Eathol |
| N6 | Ammonium nitrogen |
| N7 | Soluble non-salt solids |
| N8 | Oxalic Acid |
| N9 | Citric Acid |
| N10 | Tartaric Acid |
| N11 | L-Malic Acid |
| N12 | Succinic Acid |
| N13 | Lactic Acid |
| N14 | Acetic Acid |
| N15 | Pyroglutamic Acid |
| N16 | Asp |
| N17 | Thr |
| N18 | Ser |
| N19 | Glu |
| N20 | Gly |
| N21 | Ala |
| N22 | Val |
| N23 | Met |
| N24 | Ile |
| N25 | Leu |
| N26 | Tyr |
| N27 | Phe |
| N28 | His |
| N29 | Lys |
| N30 | Pro |
| N31 | Ethyl Acetate |
| N32 | Ethyl phenylacetate |
| N33 | 2,2,4-Trimethyl-1,3-pentanediol monoisobutyrate |
| N34 | 2-Methylbutanal |
| N35 | 3-Methylbutanal |
| N36 | 3-Furaldehyde |
| N37 | Decanal |
| N38 | Benzaldehyde |
| N39 | Phenylacetaldehyde |
| N40 | 2,3-dihydro-1H-Indene-4-carboxaldehyde |
| N41 | 3-Methyl-1-butanol |
| N42 | 1-Octen-3-ol |
| N43 | 2-Furanmethanol |
| N44 | 3-(Methylthio)-1-propanol |
| N45 | Phenylethyl Alcohol |
| N46 | 2-Octanone |
| N47 | HEMF |
| N48 | 3-Methyl-butanoic acid |
| N49 | 3-Methyl-pentanoic acid |
| N50 | 2-Methoxyphenol |
| N51 | Maltol |
| N52 | 4-Ethyl-2-methoxy-phenol |
| N53 | 4-Ethylphenol |
| N54 | 2-Methoxy-4-vinylphenol |
| N55 | 2,4-Di-tert-butylphenol |
